# First atmospheric mercury measurements at a coastal site in the Apulia region: seasonal variability and source analysis

**DOI:** 10.1007/s11356-022-20505-6

**Published:** 2022-05-11

**Authors:** Maria Martino, Antonella Tassone, Lorenzo Angiuli, Attilio Naccarato, Paolo Rosario Dambruoso, Fiorella Mazzone, Livia Trizio, Cristina Leonardi, Francesco Petracchini, Francesca Sprovieri, Nicola Pirrone, Francesco D’Amore, Mariantonia Bencardino

**Affiliations:** 1 CNR-Institute of Atmospheric Pollution Research, Rende, Italy; 2 Apulia Region Environmental Protection Agency (ARPA Puglia), Bari, Italy; 3 MiTE, Italian Ministery of Ecological Transition, Rome, Italy; 4 CNR-Institute of Atmospheric Pollution Research, Rome, Italy; 5 Department of Chemistry and Chemical Technologies, University of Calabria, Rende, Italy

**Keywords:** Gaseous Elemental Mercury (GEM), Backward trajectory, Cluster analysis, Potential Source Contribution Function (PSCF), Anthropogenic point sources, Wildfire influence

## Abstract

**Abstract:**

In the framework of the Italian Special Network for Mercury (ISNM) “Reti Speciali”, a sampling campaign to monitor atmospheric mercury (Hg) was carried out at Monte Sant’Angelo (MSA). This is a coastal monitoring station in the Apulia region, representative of the Southern Adriatic area, within the Mediterranean basin. This work presents continuous Gaseous Elemental Mercury (GEM) measurements over about three years at MSA, using the Lumex RA-915AM mercury analyzer. The aim was to obtain a dataset suitable for the analysis of Hg concentrations in terms of source and transport variation. Diurnal cycles of GEM were evaluated to observe the influence of local atmospheric temperature and wind speed on potential re-emissions from surrounding sea and soil surfaces. Data were also analyzed in terms of long-range transport, using backward trajectory cluster analysis. The spatial distribution of potential sources, contributing to higher measured GEM values, was obtained employing Potential Source Contribution Function (PSCF) statistics. The influence of major Hg anthropogenic point sources, such as mining activities and coal-fuel power plants, both regionally and continentally, from mainland Europe, was observed. The role of the vegetation GEM uptake in modulating the seasonal GEM variability was also investigated. The potential of wildfire influence over the highest detected GEM levels was further examined using active fire data and the evaluation of the vegetation dryness index during the selected episodes.

**Graphical abstract:**

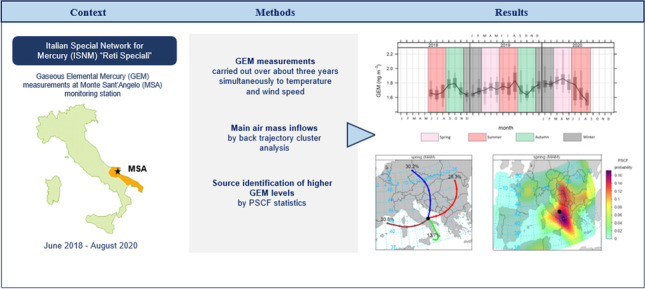

**Supplementary Information:**

The online version contains supplementary material available at 10.1007/s11356-022-20505-6.

## Introduction

Mercury (Hg) in its various forms is a pollutant with a significant impact on human health. Its numerous emission sources, its extensive use in the recent past, and historical accidents such as the Minamata disaster have over time increased the potential for human exposure to its harmful effects. This growing exposure has raised the interest of the scientific and policy community in monitoring Hg at both national and global levels. Despite continuing efforts to reduce the release of Hg into the environment, current environmental concentrations are often still of concern. In this context, UNEP (United Nations Environment Programme) promoted the realization of the Minamata Convention on Mercury (MCM) as a global commitment to protect human health and the environment from the adverse effects of Hg (UNEP 2019). In fact, Hg is a global pollutant with a complex biogeochemical cycle that represents a significant public health and environmental issue due to its toxicity, its persistence in the environment, and its ability to undergo long-distance transport before depositing to terrestrial and aquatic surfaces (Pirrone et al. 2001; Driscoll et al. 2013; Naccarato et al. 2020). Among the major sources of Hg pollution for ecosystems, the atmosphere represents the main transport (re-distribution) media of Hg in the environment (Driscoll et al. 2013) and for this reason, over the years there have been numerous studies of atmospheric Hg, with the aim of both improving knowledge in different areas of the globe (Sprovieri et al. 2016, 2017) and improving analytical performance for its monitoring (Tassone et al. 2020; Naccarato et al. 2021). In the atmosphere, Gaseous Elemental Mercury (GEM or Hg(0)) is the predominant Hg species and represents 90 to 99% of the Total Gaseous Mercury (TGM) (Sommar et al. 2020). The global environmental Hg load is due to the contribution of natural emissions from volcanoes, forest fires, sea surface and soil emissions, and the weathering of rocks, as well as anthropogenic activities such as coal combustion, cement production, oil refining, gold mining, and wastes from consumer products (Outridge et al., 2018). Fires are a major source of atmospheric Hg which transfer Hg from the terrestrial biosphere to the atmosphere by mobilizing the Hg stored in terrestrial ecosystems and directly influencing its emission and deposition cycles in the environment (e.g., in soils, surface waters, sediments, and biota) (Wiedinmyer and Friedli 2007; Bishop et al. 2020). GEM is the dominant form of Hg emitted to the atmosphere from wildfires and may be transported far from the emission sources (Wiedinmyer and Friedli 2007, De Simone et al., 2015). Anthropogenic activities are responsible for a significant proportion of global Hg input to the environment and have an impact on the health of wildlife and human populations. Anthropogenic Hg emissions come from diffuse (e.g., landfills, sewage sludge amended fields, and mine waste) and point sources, of which fossil fuel-fired power plants, cement production, mining and smelting activities, as well as incinerators, are considered to be the main anthropogenic Hg point sources (Guangliang Liu, Yong Cai 2011;Outridge et al., 2018; Charvát et al. 2020). In addition to the abovementioned geogenic and anthropogenic Hg releases, the largest emission source contributor for Hg is represented by re-emissions. This means Hg, previously deposited from the air to soils, surface waters, and vegetation from past emissions, which is emitted back into the air by processes such as evasion from water/land surfaces (Carbone et al. 2016, 2018; Ballabio et al. 2021). To improve understanding of the Hg biogeochemical cycle, several monitoring networks have been developed worldwide. The Global Mercury Observation System (GMOS) (www.gmos.eu), which is coordinated by the Institute of Atmospheric Pollution Research of the Italian National Research Council (CNR-IIA), was the first network for Hg developed on a global scale. It started in 2010 and is currently under the Global Observation System for Mercury (GOS^4^M - www.gos4m.org) GEO Flagship to support several articles for the MCM implementation (e.g., Art.22 on the effectiveness evaluation). GMOS includes monitoring sites located in both hemispheres, and in polar areas to study temporal and spatial concentration variations (Sprovieri et al. 2016, 2017). Among its more than 30 monitoring stations, the GMOS network includes two high altitude mountain observatories in Italy where atmospheric Hg, together with meteorological parameters, aerosols, and trace gases are measured continuously (Bencardino et al. 2019; Vardè et al. 2019; Barbaro et al. 2020; Moretti et al. 2021). These two Italian high altitude observatories are the Col Margherita (CMA) and the Monte Curcio (CUR) Global Atmosphere Watch (GAW) stations, located in Northern (Veneto region) and Southern (Calabria region), Italy, respectively. To increase national Hg monitoring capability, the Italian government has recently established the Italian Special Network for Mercury (ISNM) “Reti Speciali” (Italian Government 2012) which is the result of the collaboration between the CNR-IIA, the Italian National Agency for New Technologies, Energy and Sustainable Economic Development (ENEA), the Italian National Institute of Health (ISS), and the Italian Ministry of Ecological Transition (MITE). The agreement was designed to establish a “special” monitoring network that integrates the surveillance of parameters required by National and European legislation including the heavy metals included in European Directive 2004/107/EC and polycyclic aromatic hydrocarbons in ambient air. According to Art. 4 of the directive, the monitoring sites must be selected to identify the geographical variation and long-term trends of the pollutants; therefore, three background sites have been established on the Italian territory: one in the north (Schivenoglia—Lombardy region), one in the center (Montelibretti—Lazio region), and one in the south of Italy (Monte Sant'Angelo—Apulia region).

In this study, the first dataset of GEM concentrations measured at the Monte Sant’Angelo (MSA) station, measured over about three years (June 2018 to August 2020), is presented and discussed. Both the temporal and spatial variability in GEM levels were analyzed to evaluate local and long-range transport influences and to identify potential contributing sources.

## Experimental

### Sampling site description

The sampling site considered for this work is located in the municipality of Monte Sant’Angelo (MSA, 41° 39′ 55.609" N 15° 56′ 42.140" E)—the province of Foggia—in the Apulia region. It is classified as a background station in a rural area surrounded mainly by olive groves. Land cover and land use types in the area around the MSA sampling station are mainly forest, shrubland, and sparse vegetation.

**Fig. 1:**
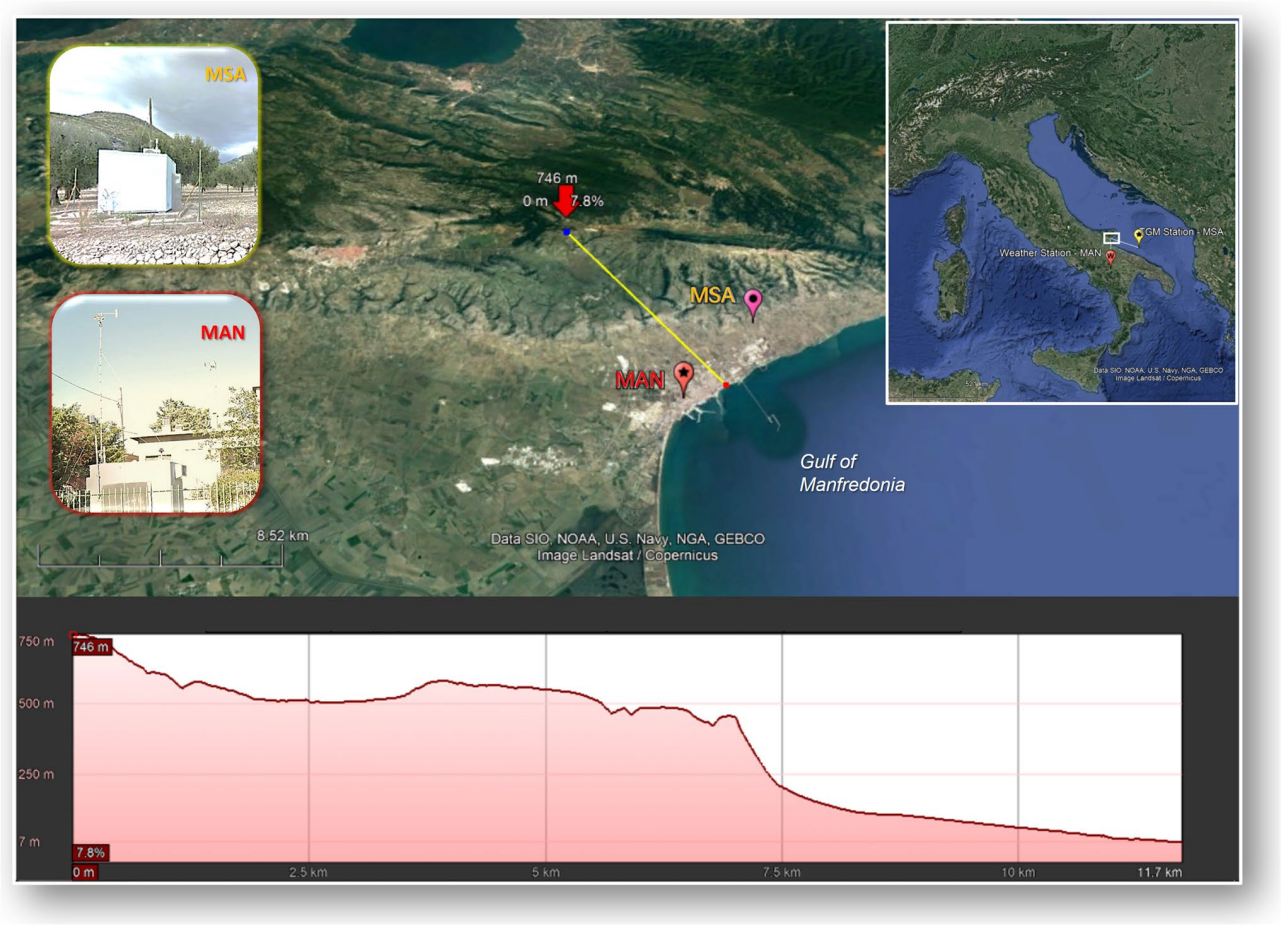
Map with altitude profile of the area surrounding the GEM sampling station (Monte Sant’Angelo—MSA) and the weather monitoring station (Manfredonia—MAN)

The station is 125 m a.s.l., on the slopes of the Gargano ridge, whose peak reaches about 800 m a.s.l. It is also considered a coastal site, given its position 2.3 km from the Adriatic Sea overlooking the Gulf of Manfredonia (see Fig. 1). As a result of its location, air masses reaching the site are influenced by winds from inland but also by sea breeze regimes. In the framework of the ISNM, the MSA station was designated as a secondary site, and therefore only GEM concentrations have been monitored.

### Atmospheric sampling campaign and GEM measurements

Atmospheric Hg concentration measurements were made using a Lumex RA-915AM automated mercury monitor, from June 2018 to August 2020. The Lumex analyzer, based on the differential atomic absorption spectroscopy with Zeeman background correction (Sholupov et al. 2004), provides direct continuous concentrations of GEM in the air flow sampled. GEM is the dominant Hg species in the background atmosphere and constitutes more than 95–98% of total gaseous mercury (TGM), which is otherwise measured through gold trap preconcentration (Sprovieri et al., 2016). Although there are operational differences in these two methods of measuring atmospheric Hg, many parallel observations with a gold trap preconcentration step and direct measurement with ZAAS confirms that the difference in results between these two methods lies within the measurement uncertainty (Brown et al. 2010, Pandey et al., 2011). The Lumex analyzer is designed for direct, long-term, non-attended Hg measurement, and allows continuous readings every second. For reporting purposes, these readings are averaged over a time interval chosen by the operator. Automatic zero drift correction and auto-calibration functions provide stable analytical parameters, operational reliability, and safety. At the MSA station, the Lumex operated with an airflow rate of 7–10 L min^−1^, a measurement resolution time of 5 s, and an acquisition averaging interval set at 60 min. The Lumex analyzer RA-915AM was automatically calibrated every 48 h and the calibration was performed with a built-in cell saturated with Hg vapor. An annual analyzer calibration was also performed in the laboratory. In addition to GEM levels, meteorological parameters as air temperature (T), wind speed (WS), and direction (WD), were acquired during the whole campaign, to characterize the different meteorological conditions of the sampling area and to investigate their influence on the seasonal and daily GEM time series. Since the MSA station is not equipped with meteorological sensors, the parameters used here are from the nearby (about 5 km) station located in Manfredonia (MAN, 41° 37′ 40.530′′ N 15° 54′ 27.310′′ E) (see Fig. 1). Both GEM data, recorded at MSA, and meteorological parameters, measured at MAN, were courteously provided by the Apulian Regional Agency for Environmental Protection and Prevention (ARPA Puglia).

### Data processing, methods, and supporting tools

The sampling campaign was carried out at MSA with the Lumex analyzer, whose measurement resolution was set to 5 s, while the program computer for data acquisition was set to 1 h of resolution. The computer program, managed by ARPA Puglia, acquires and stores only the hourly GEM data, obtained as the average of the corresponding 5-s measurements, without saving the raw dataset at their original measurement resolution. Therefore, our initial dataset consisted of GEM data with 1 h of time resolution, corresponding to the available time resolution set for data acquisition. The available hourly values of GEM concentrations were then aggregated to provide daily averages, which were only considered representative if 75% of the corresponding hourly data were available, which means at least 18 over 24 h, for each daily period. In addition, the monthly median values were calculated and only those months of the campaign had 66% of the corresponding daily averages (at least 20 days a month) were considered valid. GEM seasonal concentrations, over each available year, were also calculated for Autumn (1/9–30/11), Winter (1/12–28 or 29/2), Spring (1/3–31/5), and Summer (1/6–31/8). Seasonal median values were considered valid if at least 2 months, calculated from daily averages, were available during the period. The obtained monthly and seasonal medians were evaluated, in terms of significant differences, firstly as a whole, using the Krustal-Wallis test, and then for pairwise comparisons, with the Wilcoxon rank sum test using the adjustment method of Benjamini and Hochberg (Benjamini and Yekutieli 2001). Data tables reported in this work were set up with Microsoft Excel while data elaboration was carried out by using the *openair* package of RStudio tool, which is an Integrated Development Environment (IDE) for R, a programming language for statistical computing and graphics (Carslaw and Ropkins 2012; Carslaw 2019).

The diurnal cycle of GEM and selected meteorological parameters was assessed by considering the seasonal data available for each available year over the observation period, and by using the *timeVariation* R function. Wind rose plots were produced using the *windRose* function. To evaluate the role of vegetation GEM uptake in modulating GEM seasonality, the Normalized Difference Vegetation index (NDVI), which is a measure of the state of vegetation health based on how plants reflect light at certain wavelengths, was also considered. It is influenced by the fractional cover of the ground by vegetation, the vegetation density, and the vegetation greenness. It indicates the photosynthetic capacity of the land surface cover and is based on satellite data from the Copernicus Sentinel 2 mission (https://apps.sentinel-hub.com).

The interpretation of the GEM measurements presented in this study were also supported using various additional and complementary tools, to identify the occurrence and the impact at the MSA station of potential anthropogenic and natural sources. In particular, the Hybrid Single-Particle Lagrangian Integrated Trajectory Model (HYSPLIT) modeling, available at the NOAA Air Resources Laboratory READY Website https://www.ready.noaa.gov/HYSPLIT.php) was used to evaluate the long-range transport of air masses arriving at the monitoring site (Draxler and Rolph, 2013; Rolph 2013). Air mass backward trajectories were calculated using the *trajPlot* function of RStudio. Specifically, 72-h backward trajectories were calculated, by setting the coordinates of MSA for the receptor site, and the elevation of MSA station (125 m a.s.l.) for the trajectory’s arrival height. To group similar air mass origins, a back-trajectory cluster analysis for each available season was performed with the *trajCluster* Rstudio function. A number of 4 clusters was set for the calculation analysis. To define the individual clusters, the distance matrix, which determines the similarity (or dissimilarity) of different back-trajectories, was calculated by using the so-called “*Angle*” method instead of the simple Euclidean distance. This “*Angle*” method is a measure of how similar two back trajectory points are in terms of their angle from the origin (i.e., the starting location of the back trajectories). The angle-based measure was preferred since it often capture some of the important circulatory features in the atmosphere. The Potential Source Contribution Factor (PSCF) was also executed through ad-hoc RStudio functions allowing the combination of the GEM data with the air parcel backward trajectories. This statistical tool is generally useful to identify source areas for pollutants with a relatively long lifetime such as GEM. The PSCF is based on the HYSPLIT model and calculates the probability that a source is located at latitude *i* and longitude *j*, due to the passing of the air mass parcel. The PSCF is defined as follows:

$$\mathrm{PSCF}=\frac{m_{ij}}{n_{ij}}$$

where n*ij* is the number of times that the trajectories passed through the cell (*i,j*) and m*ij* is the number of times that a source concentration was high when the trajectories passed through the cell (*i, j*). In this study, PSCF was applied to identify the probability of a grid cell being associated with high GEM events and using a concentration cut-off criterion value set at the 90^th^ percentile. Over the entire dataset, 3-day backward trajectories were generated, and the corresponding GEM hourly values were associated with the trajectory. For the PSCF maps, a domain including the Mediterranean basin and the European continent (35°–50° N; 10° W–30° E) was considered with at 1°×1° resolution using the Lambert conformal projection (Carslaw 2015). Lastly, in order to identify the location of each individual fire hotspot that occurred in areas surrounding the MSA monitoring station, the Fire Information for Resource Management System (FIRMS) was used ((Firms-fire information for resource management system n.d.)—https://earthdata.nasa.gov/firms). To corroborate the vegetation dryness, during the identified episodes, the Normalized Difference Moisture Index (NDMI), from the Copernicus Sentinel-2 mission, which provides data concerning vegetation water content, for drought monitoring (https://apps.sentinel-hub.com), was considered.

## Measurement results and discussion

### Variability of GEM levels on hourly and daily basis

**Fig. 2:**
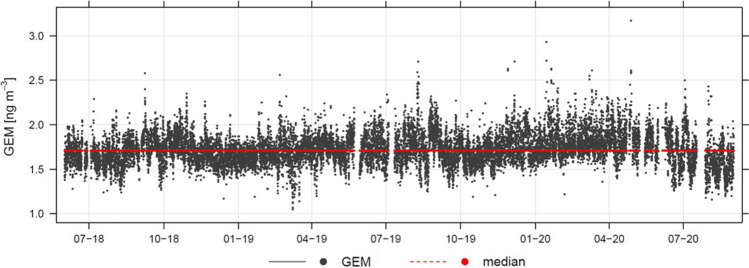
Hourly GEM concentrations at MSA station. The median value, detected throughout the whole sampling campaign, is also reported

The GEM dataset consisted of 17987 hourly GEM data, from June 2018 to August 2020 (see Fig. 2). This dataset is stored in the dedicated database of the Reti Speciali Network, accessible with credentials from its reference web portal (www.retispeciali.it). As can be seen in Fig. 2, the available GEM dataset presented some interruptions, some due to the instrument calibration or maintenance. Over the whole sampling period at the MSA site, the atmospheric GEM variability showed hourly concentrations ranging from 1.05 to 3.17 with a median value of 1.71 ng m^−3^. In terms of daily averaged GEM levels, we obtained 784 valid daily averaged GEM data, varying from 1.32 to 2.39 with a median value of 1.72 ng m^−3^ and a mean value of 1.73 ng m^−3^. The mean value found at MSA over the entire measurement period calculated from the daily GEM averages is perfectly in line with concentrations typically found in the Mediterranean area (Hedgecock et al. 2003; Pirrone et al. 2003; Sprovieri et al. 2003, 2010; Sprovieri and Pirrone 2008; Kotnik et al. 2014). Even if obtained with different methods, no difference exists between TGM and GEM from the point of view of the obtained measurement data (Brown et al. 2010; Sprovieri et al. 2016). Therefore, we compared the GEM levels recorded at MSA, through the Lumex analyzer, with those from other sites of the Mediterranean basin, mainly recorded with the Tekran mercury analyzer based on gold trap preconcentration (Tekran Instrument Corp., Ontario, Canada). During the Medoceanor cruises, performed from 2000 to 2006 in the Mediterranean Eastern sector, with reference to the over waters measurements, the GEM mean values over each sampling period, calculated from daily GEM averages, varied in fact between 1.20 and 2.00 ng m^−3^ (Sprovieri et al., 2010). The range of daily averaged GEM levels at MSA compared with the measurements performed at selected sites distributed throughout the Mediterranean basin within both the EU funded MAMCS and MERCYMS research projects (Pirrone et al. 2003), shows also a similar range of daily averaged values among all sites (Wängberg et al. 2008).

A monitoring site in Greece also showed atmospheric Hg values consistent with that observed at MSA. This site is not affected by local sources, as highlighted by the daily GEM averaged concentrations which remained quite constant for long periods (less than 1.50 ng m^−3^), while high daily averaged values were only due to occasional anthropogenic emissions from land (Polyzou et al. 2021).

### Variability of GEM on monthly basis

**Fig. 3:**
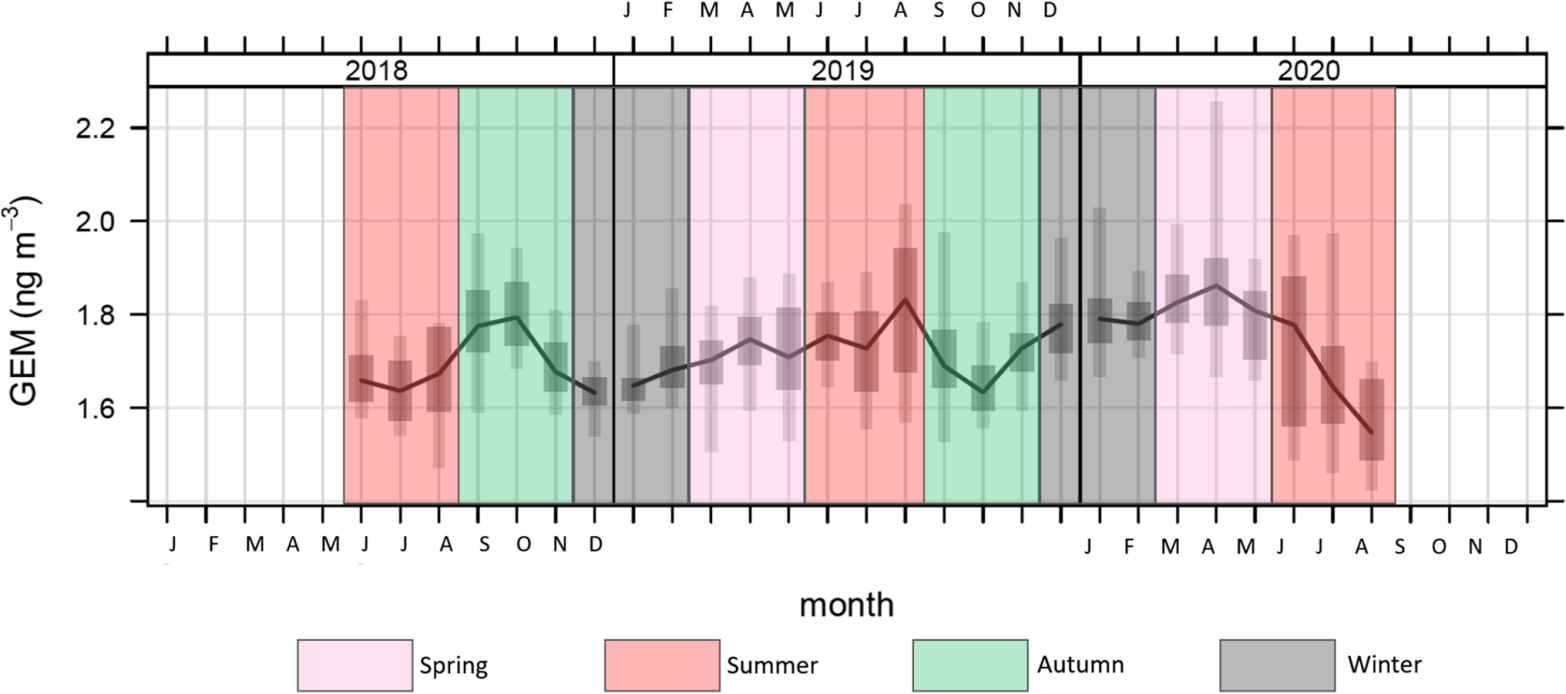
Box-and-whisker plot, showing the distribution, on monthly basis, of GEM concentrations observed during the whole sampling campaign at MSA. For each month, the bold line indicates the median, the bottom, and top edges of the box indicate the 25/75^th^ percentiles, while the bottom and top whiskers refer to the 5/95^th^ percentiles. Monthly distributions are further grouped by shaded areas, whose colors refer to the corresponding season

The statistical distribution of monthly GEM values is shown in the box-plot graph (Fig. 3) showing that the behavior of GEM was different through the months over the 3 years of our observations. As also confirmed by the Kruskal-Wallis rank sum test (chi-squared = 271.37, *p*-value < 2.2e-16), there were significant differences between the GEM monthly values observed at MSA, whose pairwise comparisons were carried out by using the Wilcoxon rank sum test. From the output of the Wilcoxon test, whose results are summarized in Table S1, for 2018, the GEM median value measured in October (1.81 ng m^−3^) was found to be statistically similar (*p*>0.05) to that recorded in September (1.77 ng m^−3^). However, GEM levels during these two months were both found to be statistically higher (*p*< 0.05) than those recorded in the other months of 2018 (see Table 1). During 2019, the highest median value was recorded in August (1.79 ng m^−3^), with little statistical difference (*p*>0.05) with respect to April, May, June, July, and December, but significantly higher (*p*<0.05) in comparison to January, February, March, September, October, and November (see Table 1 and Table S1). In 2020, the highest GEM median value was recorded in April (1.83 ng m^−3^), which was statistically similar (*p*>0.05) to January (1.78 ng m^−3^) and March (1.80 ng m^−3^), while it was significantly higher (*p*<0.05) than the median values obtained for the remaining months of the year (see Table 1 and Table S1). The observed differences in GEM levels recorded over the available years will be discussed in terms of seasonal variability in the following section.

**Table 1:** Monthly median GEM values and, in brackets, the number of corresponding available daily means. In bold are evidenced the median values similarly significantly higher than the other ones, as resulting from the Wilcoxon test (see Table S2)

**Month -Year**	**2018**	**2019**	**2020**
	GEM (ng m^−3^)	GEM (ng m^−3^)	GEM (ng m^−3^)
January	-	1.66 (*31*)	**1.78** (*31*)
February	-	1.68 (*28*)	1.76 (*28*)
March	-	1.72 (*31*)	**1.80** (*31*)
April	-	**1.74** (*30*)	**1.83** (*28*)
May	-	**1.71** (*26*)	1.82 (*25*)
June	1.67 (*28*)	**1.74** (*30*)	1.72 (*26*)
July	1.63 (*28*)	**1.73** (*25*)	1.69 (*22*)
August	1.66 (*31*)	**1.79** (*31*)	1.56 (31)
September	**1.77** (29)	1.71 (*30*)	-
October	**1.81** (*31*)	1.63 (*31*)	-
November	1.71 (*30*)	1.73 (*30*)	-
December	1.60 (*31*)	**1.78** (*31*)	-

### Seasonal variation and meteorological parameters

When analyzed on a seasonal basis, GEM levels recorded at MSA showed an overall seasonal variability confirmed by the Kruskal-Wallis rank sum test (chi-squared = 204.01, *p*-value < 2.2e-16) while the pairwise comparisons between each season, made by applying the Wilcoxon rank sum test, gave the paired *p*-values summarized in Table S2 of the Supplementary Information. Outcomes from this test proved that, compared to all the other available seasons, the significantly highest value was recorded in spring 2020 (1.83 ng m^−3^) (see Table 2). Comparing the same seasons over the different available years, apart from spring 2020, we also found that autumn 2018 (1.76 ng m^−3^) was significantly higher (*p*<0.05) than autumn 2019 (1.69 ng m^−3^). Regarding the available summer seasons, the significantly higher value resulted for summer 2019 (1.75 ng m^−3^) if compared to summer 2018 and 2019 (1.66 and 1.64 ng m^−3^, respectively, with both pairs *p*-values<0.05). For winter, the same comparative test showed greater values for winter 2020 with respect to winter 2019 (1.77 and 1.63 ng m^−3^, respectively with pairs *p*-value<0.05). Seasonal variations in GEM concentrations have been described at other European sites (Sprovieri et al., 2016; Jiskra et al., 2018). Usually, GEM concentration peaks in winter while the minimum is observed in late summer/early autumn, similar to observations at MSA in 2020. This seasonality is widely recognized to be mainly driven by two factors: the higher emissions from coal combustion, due to higher energy demands for heating during colder months (Weigelt et al., 2015), and the higher GEM oxidation rate during warmer months (Selin et al., 2007; Horowitz et al., 2017). Recently, the role of vegetation GEM uptake in modulating its seasonality was additional investigated as a cofounding factor (Jiskra et al., 2018). The vegetation uptake seems in fact to be responsible for GEM depletion when the vegetation activity is high. In this regard, for the area around the MSA sampling site, we looked at the NDVI, which is a simple but effective index for quantifying green vegetation. The NDVI values observed during each seasonal period, for which GEM concentrations are available at MSA, are reported in Fig. S1. It is possible to notice that during winter 2019 the NDVI values were higher over the investigation area, in respect to those observed in 2020, during which the recorded NDVI values were close to zero, probably corresponding to snow on the Gargano ridge. This evidence could be the raison explaining the difference between lower GEM concentrations recorded in winter 2019 (1.63 ng m^−3^), in respect to those observed in winter 2020 (1.77 ng m^−3^). Similarly, the map with NDVI values during autumn 2018 show widespread areas around the MSA sampling station with values close to zero, in this case probably corresponding to drier areas without vegetation. This evidence may also explain the difference in GEM concentrations between its levels recorded in autumn 2018 and autumn 2019. The higher vegetation activity observed in autumn 2019 may have contributed to a greater uptake of GEM by the vegetation itself, resulting in the lower GEM values measured during this season (1.69 ng m^−3^) compared to those observed in 2018 (1.76 ng m^−3^). For spring and summer seasons, there was not a net difference noticeable from the NDVI maps through the investigated years. However, the differences in GEM levels, observed between summer and spring seasons over the available years, may be attributed to the temperature-dependence of GEM re-emissions, from both soil and sea surfaces (Baya ansd Van Heyst, 2010). While the intensity of wind did not show any relevant seasonal variations, the median temperature values at MSA show the same seasonal differences recorded in terms of median GEM concentrations (see Table 2).

**Table 2:** Seasonal median values for GEM (ng m^–3^), T (°C), and WS (m s^–1^). In brackets, the number of corresponding available daily means

**Season to year**	**2018**	**2019**	**2020**
	**GEM (ng m**^**−3**^)	**GEM (ng m**^**−3**^)	**GEM (ng m**^**−3**^)
Winter	-	1.63 (*90*)	1.77 (*90*)
Spring	-	1.72 (*87*)	1.83 (*84*)
Summer	1.66 (*87*)	1.75 (*86*)	1.64 (*79*)
Autumn	1.76 (*90*)	1.69 (*91*)	-
	**T (°C)**	**T** **(**°**C)**	** T (°C)**
**Season to year**	**2018**	**2019**	**2020**
Winter	-	9.0 (*90*)	10.6 (*91*)
Spring	-	15.0 (*92*)	15.8 (*92*)
Summer	26.9 (*92*)	27.5 (*92*)	26.3 (*92*)
Autumn	19.4 (*91*)	19.0 (*91*)	-
	**WS (m s**^**–1**^)	**WS** **(m/s)c**	**WS** **(m/s)**
**Season to year**	**2018**	**2019**	**2020**
Winter	-	0.8 (*90*)	0.7 (*91*)
Spring	-	1.0 (*92*)	1.0 (*92*)
Summer	1.1 (*92*)	0.9 (*92*)	1.0 (*92*)
Autumn	0.8 (*91*)	0.7 (*91*)	-

**Fig. 4:**
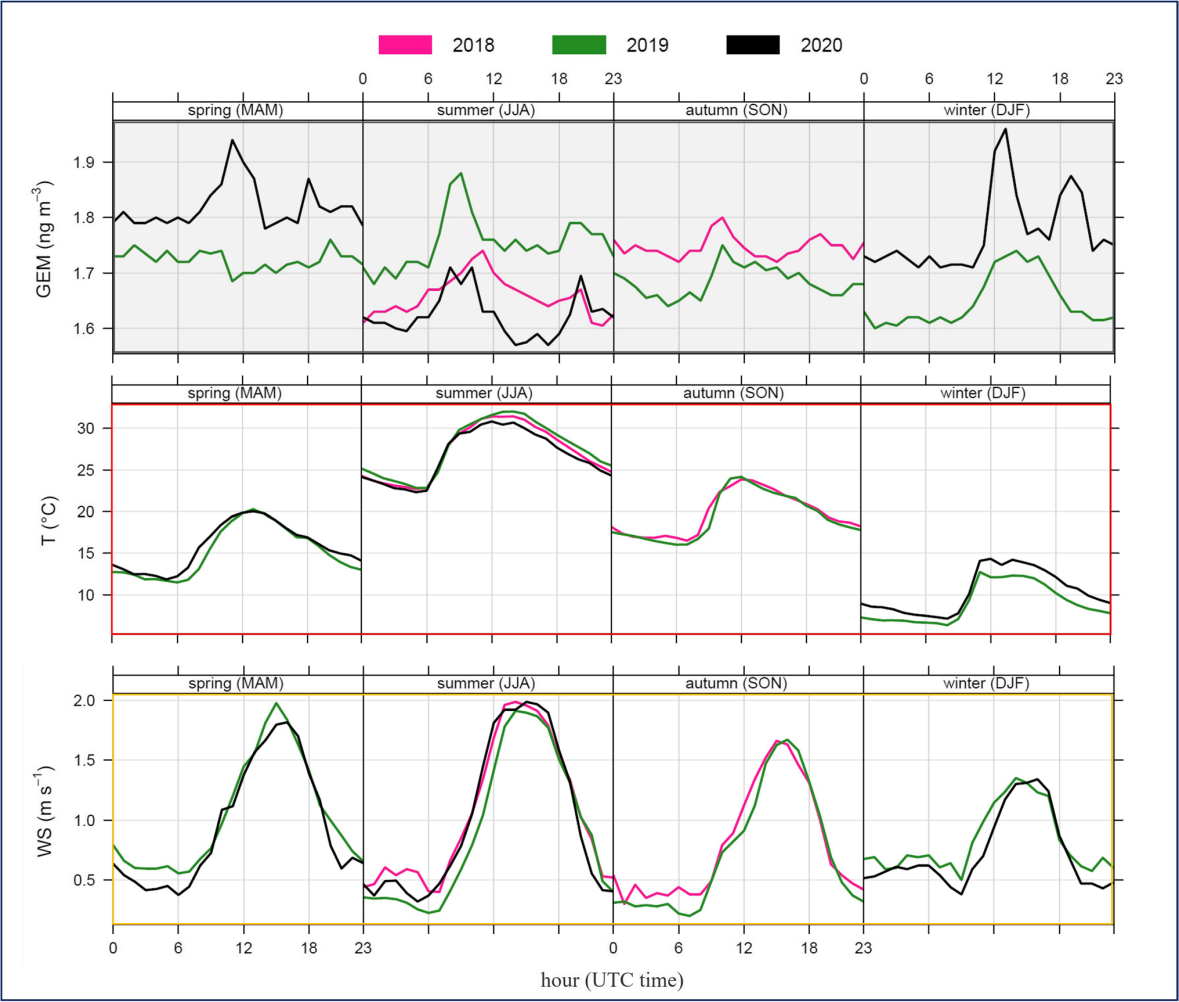
Diurnal cycle, by seasons and by years, for median values of GEM (ng m^–3^) and selected meteorological parameters, T (°C) – Temperature, and WS (m s^–1^) – Wind Speed, with reference to the whole sampling campaign at MSA

These outcomes for the GEM seasonal variation over each of the available years are also evident in Fig. 4, where the GEM seasonal diurnal cycles by year, together with the simultaneously recorded temperature and wind speed parameters, are further reported.

As the figure makes clear, the median diurnal cycle of GEM concentrations and meteorological parameters showed a similar pattern over all the available seasons. It can be observed that generally the GEM levels started to increase early in the morning, gradually reaching highest values before midday (UTC time) in correspondence of an increase in the hourly median values of temperature. The following increase in wind speed determine a drop in GEM values, which rise again in the afternoon, reaching a second lower peak, finally followed by a slow decline in concentrations overnight. The increases in GEM levels, observed late in morning and during afternoon, both occurred during the daily hour with higher temperatures. Therefore, these increases could be related to those mechanisms allowing the potential Hg re-emissions deriving from the soil and vegetation surfaces (Gworek et al. 2017; Ma et al. 2018). As observed elsewhere (Ci et al. 2011; Lan et al. 2012; Fu et al. 2015; Diéguez et al. 2019), the observed GEM diurnal behavior would also reflect the re-emission from the sea surface enhanced by warmer temperatures and higher solar radiation levels, which in turn promote Hg photo-reduction (Soerensen et al. 2010; Castagna et al. 2018). The local wind direction is influenced by the complex orography of the area, characterized by the proximity to the sea surface and by the presence of the Gargano ridge with a significant slope (see Fig.1). As is clear in Fig. S2, where the wind roses, with the wind speed/direction frequencies, are reported, by each season over the available years, the two prevailing local wind directions were from the south-south-east and north-west. The GEM behavior described above was similarly observed during each season with a difference noticed for winter, when the GEM peak was recorded after midday (UTC time) instead of the late morning. The reason may be attributed to the weaker solar radiation during the winter season. It is also worth noting that, differently to the other seasons, during winter there was a net WD prevalence from the south-easterly sector with associated higher WS values. The winter GEM peaks were predominantly recorded in correspondence of air masses coming from sea and likely associated not only to the re-emission from the sea surface but also with long-range transport. This possibility requires further analysis supported by backward trajectories, as discussed in the following sections.

### Mercury source attribution via HYSPLIT modeling

#### Back trajectory cluster analysis for MSA station

**Fig. 5:**
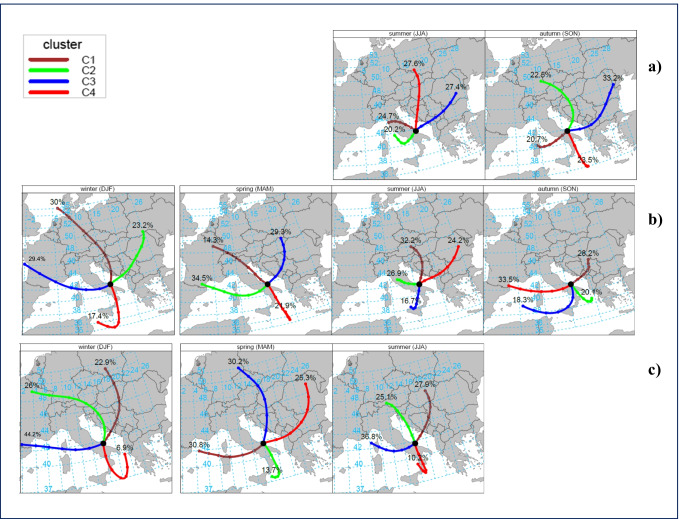
Cluster analysis, on seasonal basis, considering runs of 72-h back-trajectories with MSA as receptor site, and executed over each available year of observation: **a)** 2018, **b)** 2019, and **c)** 2020. For each season, the 4-cluster solution shows the mean trajectory for each cluster

A further analysis was performed to visualize the long-range transport of air masses to MSA station and track the potential sources of GEM. The back-trajectories, generated by HYSPLIT, were managed using specific functions of the R package “openair.” Three days (72-h) back-trajectories were imported through the *importTraj* function, with settings specific to our receptor site, and then used as input to the *trajCluster* function. A cluster analysis of the back- trajectories was then executed, on a seasonal basis and over the three available years of our observations, to group similar air mass origins. The results, Fig. 5, permit the visualization of the mean trajectory for each of the identified 4 clusters, the sector of origin, their frequency, and how they changed from season to season throughout the whole sampling period at MSA.

Above all, the overview in Fig. 5 allowed us to observe that the long-range transport, although it was present in all seasons, originated from more distant areas during both the winter and spring seasons, from intermediate distances during autumn, while trajectories were rather brief in summer, probably due to the occurrence of stable summer meteorological conditions over the Mediterranean basin. The air mass inflows during summer 2018–2019 featured by two different clusters, both originating in proximity to Italian territory, with a total contribution of about 50%, thus corroborating the possibility of the influence of a local source. The proximity of a source origin was also observed in summer 2020, spring 2019–2020, and autumn 2018–2019, however in these latter cases, associated with only one cluster accounting for around 10–24% of the trajectory frequency and with a pronounced south-easterly direction of provenience. Apart from the regional origin characterization already mentioned, it was possible to identify another common pattern from the back-trajectory air mass origin map. There was, in fact, a common cluster originating from the western part of the Mediterranean basin that, with the exceptions of summer 2018 and 2019, accounted for more than 30% of trajectory frequency in all the other seasons. Another pattern that was observed to be common to all the seasons analyzed, with the exception of autumn 2019, concerned two different clusters both originating from north-eastern Europe, and showing a combined contribution of almost 50%. This sector of origin (N-E) was therefore confirmed as one of the main directions of provenance for air masses collected at MSA. Among these clusters, whose air masses origin comes from the northern-eastern sectors, it is also noteworthy the shape of line associated with their mean trajectory. For spring and winter 2020, it is possible to recognize two cluster lines with a well-defined clockwise-type sweep consistent with air masses associated with a high-pressure system. The presence of an anticyclone determines atmospheric synoptic conditions usually favoring the accumulation and limiting the dispersion of atmospheric pollutants. This the reason that probably contributed to determine the highest GEM concentrations, exactly observed during spring and winter 2020 seasons (see Table 2). Otherwise, only in the season in which the lowest GEM concentrations were recorded (winter 2019), it is possible to notice the presence of a cluster whose air masses originate from the North Sea and then follow a trajectory at high altitude, thus contributing with a cleaner air inflow towards the sampling site.

#### Source identification of higher GEM levels at MSA by PSCF statistics

With the aim to identify the potential, regional or interregional, source areas affecting higher GEM levels at MSA, the HYSPLYT back-trajectories were associated with the available GEM dataset, and then plotted on a map, using the Potential Source Contribution Function (PSCF). Modeling used in conjunction with atmospheric pollutant concentrations measured at a receptor site are commonly referred to as Trajectory Statistical Methods (TSM) (Kabashnikov et al. 2011), whose application reported in literature also includes various studies on atmospheric mercury (Kabashnikov et al. 2011; Weiss-Penzias et al. 2011; Diéguez et al. 2019). A TSM model involves counting the frequency of the back-trajectory segment endpoints in grid cells that make up the geographical domain of interest for the receptor site. The PSCF applied in this work shows the percentage frequency of trajectory segment endpoints above a concentration threshold (set at the 90^th^ percentile) relative to the total trajectory segment endpoints in each grid cell. A separate PSCF analysis was performed, on a seasonal basis, on the 2018, 2019, and 2020 datasets. The maps obtained are shown in Fig. 7, where the source areas contributing to the higher GEM levels recorded at MSA, are highlighted. The color scale is a dimensionless probability scale, which indicates the likelihood of a source region contributing to higher (>90^th^) GEM levels measured at MSA. It can be seen that the source areas contributing most are associated with a higher probability during those seasons for which the significantly highest (spring 2020) and the similarly higher (autumn 2018, summer 2019, and winter 2020) median GEM values were identified (see Table 2). To characterize these hot-spot source areas, an investigation was carried out at European level, with specific regard to those anthropogenic activities associated with larger Hg emissions, such as historical mining areas and coal-fired power plants. As reported by Ballabio et al. (2021) and illustrated in Fig. S3, in Europe there are still 87 hotspots with known mining sites, the most important of which are the Idrija mine in Slovenia (Gosar et al. 2016) the Almadén mine in Spain (Millán et al. 2006) and the Monte Amiata (Rimondi et al. 2015). Even in the case of closure of these activities, re-emission of legacy mercury from soil areas surrounding these mining sites, still represent strong localized emission sources of atmospheric GEM (Ferrara et al. 2000; Zhu et al. 2018). Regarding coal combustion in power plants, as reported by the European Union Office, in 2020 there were 166 coal-fired power plants operating in 18 EU countries, with a total capacity of 112 GW (Kapetaki et al. 2021). As can be seen in the map in Fig. S4 (Kapetaki et al. 2021), the highest density of European coal power plants lies in an area stretching from the Netherlands, across Germany and the Czech Republic, to Poland, Romania, and Bulgaria. As Fig. S5 shows, in addition to power plants located in the EU countries, there are about 16 additional polluting lignite coal power plants in Western Balkan countries (Bosnia and Herzegovina, Macedonia, Montenegro, Kosovo, and Serbia) that on average emit 20 times more sulfur dioxide (SO_2_) and 16 times more particulate matter (PM) than the average for European plants (Jensen 2019).

**Fig. 6:**
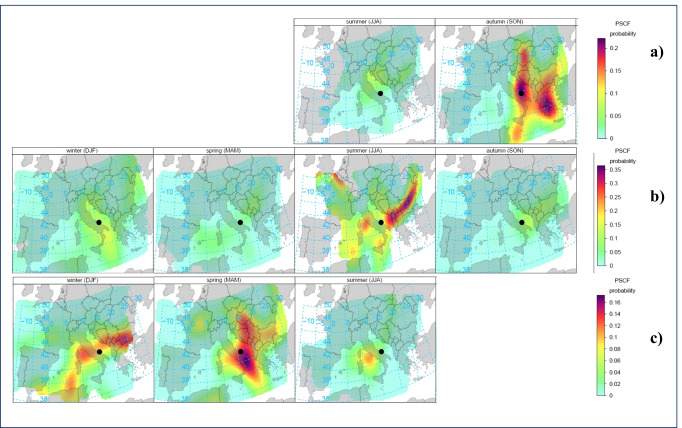
Gridded and smoothed PSCF probabilities for GEM concentrations (90^th^ percentile) carried out for each available year of observation: **a)** 2018, **b)** 2019, and **c)** 2020

With the abovementioned maps in mind and looking at the results from the application of PSCF statistics, it was possible to identify some potential key sources with a high probability of contributing to the higher GEM levels recorded at the MSA station. Overall, we can observe that the prevailing north-easterly and easterly air masses, identified with the previous back-trajectory cluster analysis, make it possible for MSA to intercept potential mercury emissions from the coal power plants located in eastern Europe, in correspondence with the areas highlighted in Fig. 6. In particular, we identified the contribution of countries such as Slovakia, Hungary, and Bosnia, in autumn 2018, Bulgaria and Republic of North Macedonia, in summer 2019, Bulgaria, Serbia and Montenegro, in winter 2020, and Bosnia, Serbia, and Bulgaria during spring 2020. The PSCF results further demonstrated an important contribution during autumn 2018, originating from the Peloponnese region, in Greece, precisely where a power plant that produces electricity from coal and lignite is located. During autumn 2018, it was possible to recognize a contribution originating within the surrounding area of the sampling site location. Otherwise, during spring 2020, it was possible to recognize a higher probability source contribution located around the south-east of the Apulia region, where the Federico II 2640 MW capacity coal-fired power plant is located. Furthermore, from the PSCF maps, it is possible to notice, with a lower probability, a common contributing source area for both summer 2019 and winter and spring 2020, coinciding with the Monte Amiata region, whose surrounding soil area continues to be contaminated by Hg from former mining activity (see Fig. S3). In proximity to this source area, there is also the Torrevaldaliga Nord power plant, which is another important coal-fired thermoelectric power plant, with a total installed capacity of 1980 MW. In addition to the above-described anthropogenic activities, the biomass burning influence was also investigated. The FIRMS maps summarized in Fig. S6-8 show a high density of active hot-spot fires, over the Western Balkan and the Eastern European countries, as well as the Southern Italy, through all seasons except for winter. Therefore, as resulted by the PSFC analysis (Fig. 6), the higher GEM levels observed during autumn 2018, summer 2019, and spring 2020 could be also affected by biomass burning emissions. Differently, there is not the same evidence for winter 2020 (see Fig. S8), for which we can confidently exclude a wildfire emission contribution to the higher GEM concentrations observed during this season at MSA.

#### Identification of sources during specific highest GEM episodes

**Fig. 7:**
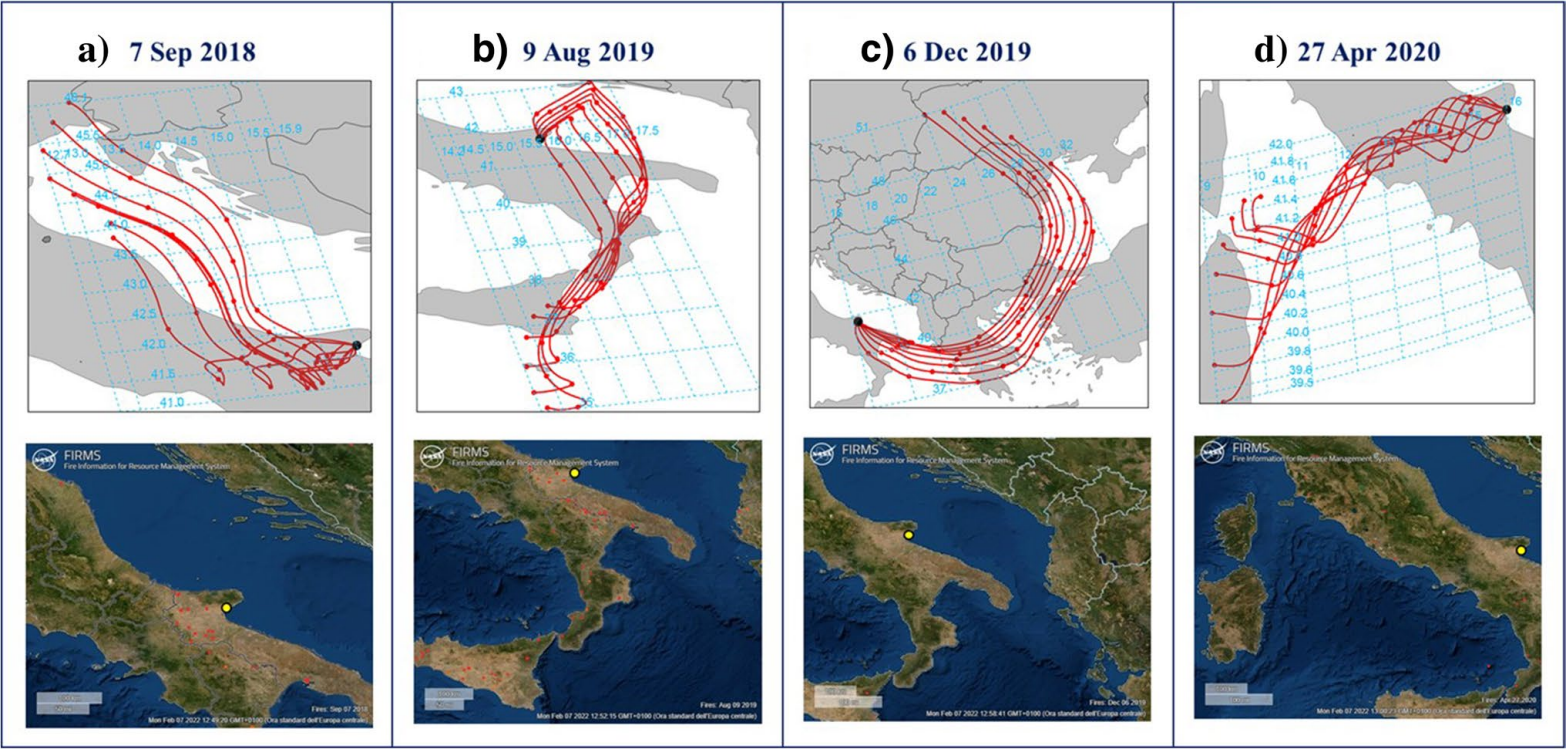
72-h backward trajectory and FIRMS maps showing location of active hot-spot fires within the surrounding area of MSA station for the following identified highest GEM episodes: **a)** September 7, 2018, **b)** August 9, 2019, **c)** December 6, 2019, and **d)** April 27, 202

To further examine specific high GEM episodes, we calculated the maximum daily values for those seasons associated with significantly higher GEM values (autumn 2018, summer 2019, winter 2020, and spring 2020). Over these seasons, we found the following highest daily GEM values, measured on (a) September 7, 2018 (2.28 ng m^−3^); (b) August 9, 2019 (2.19 ng m^−3^); (c) December 6, 2019 (2.06 ng m^−3^); and (d) April 27 2020 (2.39 ng m^−3^). HYSPLIT modeling through R studio was executed to run 72-h back-trajectories, with MSA station as the receptor site, and for each of these dates. Air mass origins and pathways, illustrated in Fig. 7 (upper panels), were completely different from each other, however confirming the greater winter influence of long-range transport with respect to the other seasons. To identify the sources for the highest GEM episodes, we further checked the potential influence of wildfire events, whose associated Hg emissions are estimated to be notable (Friedli et al. 2009; Webster et al. 2016). Therefore, the FIRMS hot-spot wildfire occurrences, within the surrounding areas intercepted by the back-trajectories, were verified for each high GEM episode (see Fig. 7 lower panels). To corroborate the vegetation dryness, during these episodes, the Sentinel-2 based Normalized Difference Moisture Index (NDMI), by, which provides an indication of vegetation water stress, was checked and the related maps are summarized in Fig. S9. The results highlighted the almost complete absence of fires during the winter episode (December 6, 2019) and the spring one (April 27, 2020). The corresponding FIRMS maps (Fig. 7 c, d—downside) do not show any fire hotspots, while the NMDI maps (Fig. S9 c, d), showed a prevalence of blue colored pixels with values ranging from 0.4–1, which correspond to vegetation without water stress. Therefore, for both the winter and spring highest GEM episodes, we can exclude the wildfire influence and consider the prevalent contribution from anthropogenic point sources. The episode detected on December 6, 2019 was in fact characterized by a 72-h back-trajectory originating from Ukraine/Romania. This feature suggests that on this day, the highest GEM value recorded at MSA could be influenced by the greater Hg emissions originating, as discussed in the previous paragraph, from various coal power plants located in eastern Europe. In this specific episode, the permanence of air masses over the southern part of the Apulia region, a few hours before their arrival at the MSA receptor site, could imply an important additional contribution from the Federico II power plant, which is one of Europe’s largest coal-fired power plants, located at Brindisi, in the Apulia region, very close to the MSA station. For the spring episode, the back-trajectory path (Fig. 7 d-upper panel) suggests a potential influence related to both sea and soil re-emissions of Hg, as the intercepted areas include the historical Hg mining district of Monte Amiata (see Fig. S3) (Vaselli et al. 2013; Ballabio et al. 2021; Fornasaro et al. 2022). The remaining autumn (September 7, 2018) and summer (August 9, 2019) episodes were differently characterized by back-trajectories associated with shorter-range transport, with a local/regional origin, and in both cases, with FIRMS maps give evidence, that during these days, there were numerous active fires in the area surrounding the MSA station (see Fig. 7 a, b-lower panels). For these two episodes, in corresponding with those areas intercepted by back-trajectories before arriving at the monitoring station, the NDMI values were found to be negative, thus indicating dry vegetation, subjected to water stress (Fig. S9 a, b). Therefore, for these episodes, we can confidently identify the Hg emissions from vegetation and soil burnt by fires, as a major contributing natural source for the highest autumn and summer GEM episodes detected at MSA. For the event with high GEM concentration recorded on August 9, 2019, we can additionally take in consideration the contribution from the volcanic Etna degassing activity (https://www.ct.ingv.it/). As confirmed by Fig. 7b (upper panel), the 72-h backward trajectories, before to arrive at MSA, passed over the eastern Sicily, where the air masses may have intercepted the plume emission fallout from Etna.

## Conclusions

A first assessment of GEM concentrations at the MSA background monitoring station, located in a rural/coastal site of the Apulia region, Southern Italy, was provided in this work. The measurement campaign was carried out from June 2018 to August 2020, during which meteorological parameters were examined to explore the diurnal cycle variability of GEM. Results, in line with other works, showed increasing GEM concentrations with concomitant higher values of temperature and wind, corroborating as potential local sources the GEM re-emissions from the surrounding sea and land surfaces, related in both cases to photo-reduction mechanisms. Long-range transport, as shown by a backward trajectory cluster analysis, was more important in winter, with a lesser influence during summer seasons. The impact of potential, regional or interregional, Hg source areas contributing with greater probability to the higher GEM levels recorded at our site at MSA station, was evaluated using PSCF statistics, associating the HYSPLIT 72-h back-trajectories with the available GEM dataset. Coal power plants, densely distributed over the north-eastern Europe and over the western Balkan countries, were identified as one of the main contributing anthropogenic sources when coupled with long-range transport. On a regional scale, over the Italian territory, the nearby Federico II coal-fired power plant, located in the south-east of the Apulia region, and the Monte Amiata area in Tuscany, highly contaminated by past mining activities, were demonstrated to be potentially significant contributors to some of the highest GEM episodes. Considering how wildfire events can impact atmospheric Hg, their influence was also investigated during specific high GEM episodes. The FIRMS tool, showing the occurrence of wildfire hotspots, coupled with NDMI maps, as provided by Copernicus Sentinel-2, showed that two out of four of the highest GEM episodes at MSA were associated with emissions from vegetation and soil during fires. Volcanic degassing emissions from Etna may also have contributed when the air masses originated over the eastern Sicily. Overall, outcomes highlighted in this study provided a preliminary overview on the main mechanisms and factors influencing the variability of GEM concentrations at one of the only five air quality stations in Italy equipped to monitor atmospheric Hg levels.

The MSA sampling station is still continuing to measure GEM levels together with other key atmospheric parameters. Their further investigation will add more elements to the first assessment herein discussed, thus providing more knowledge on Hg fate and transport around southern Italy.

## Supplementary Information

ESM 1 (DOCX 17.5 MB)

## Data Availability

Data and materials are available through the web portal www.retispeciali.it
