# Supplementary material for: First atmospheric mercury measurements at a coastal site in the Apulia region: seasonal variability and source analysis
Source: Environ Sci Pollut Res Int. 2022 May 11;29(45):68460–75. doi: 10.1007/s11356-022-20505-6 (PMC9508219; doi:10.1007/s11356-022-20505-6)
Supplement: Supplementary file 1 — (DOCX 17.5 MB) [file 11356_2022_20505_MOESM1_ESM.docx]

**Supplementary Information**

- - 1. **First atmospheric mercury measurements at a coastal site in the Apulia Region:**
    2. **seasonal variability and source analysis**

Maria Martino^1^, Antonella Tassone^1^, Lorenzo Angiuli^2^, Attilio Naccarato^1,5^,

Paolo Rosario Dambruoso^2^, Fiorella Mazzone^2^, Livia Trizio^2^,

Cristina Leonardi^3^, Francesco Petracchini^4^, Francesca Sprovieri^1^, Nicola Pirrone^1^,

Francesco D’Amore^1^, Mariantonia Bencardino^1*^

*^1^ CNR-Institute of Atmospheric Pollution Research, Division of Rende, Italy*

*^2^* *Apulia Region Environmental Protection Agency (ARPA Puglia), Bari, Italy*

*^3^ MiTE, Italian Ministery of Ecological Transition, Rome, Italy*

*^4^ CNR-Institute of Atmospheric Pollution Research, Rome, Italy*

*^5^ Department of Chemistry and Chemical Technologies, University of Calabria, Rende, Italy*

**Corresponding author e-mail address* [*bencardino@iia.cnr.it*](mailto:bencardino@iia.cnr.it)

|  | **apr_19** | **apr_20** | **aug_18** | **aug_19** | **aug_20** | **dec_18** | **dec_19** | **feb_19** | **feb_20** | **jan_19** | **jan_20** | **jul_18** | **jul_19** | **jul_20** | **jun_18** | **jun_19** | **jun_20** | **mar_19** | **mar_20** | **may_19** | **may_20** | **nov_18** | **nov_19** | **oct_18** | **oct_19** | **sep_19** |
| --- | --- | --- | --- | --- | --- | --- | --- | --- | --- | --- | --- | --- | --- | --- | --- | --- | --- | --- | --- | --- | --- | --- | --- | --- | --- | --- |
| **apr_20** | **4.6E-04** | - | - | - | - | - | - | - | - | - | - | - | - | - | - | - | - | - | - | - | - | - | - | - | - | - |
| **aug_18** | **1.2E-02** | **2.3E-06** | - | - | - | - | - | - | - | - | - | - | - | - | - | - | - | - | - | - | - | - | - | - | - | - |
| **aug_19** | 9.1E-02 | 3.0E-01 | **1.8E-03** | - | - | - | - | - | - | - | - | - | - | - | - | - | - | - | - | - | - | - | - | - | - | - |
| **aug_20** | **5.9E-07** | **2.4E-08** | **3.0E-03** | **2.1E-06** | - | - | - | - | - | - | - | - | - | - | - | - | - | - | - | - | - | - | - | - | - | - |
| **dec_18** | **4.9E-06** | **9.5E-08** | 1.0E-01 | **4.4E-04** | **3.4E-02** | - | - | - | - | - | - | - | - | - | - | - | - | - | - | - | - | - | - | - | - | - |
| **dec_19** | 2.1E-01 | **2.4E-02** | **3.0E-04** | 5.2E-01 | **4.2E-08** | **6.4E-08** | - | - | - | - | - | - | - | - | - | - | - | - | - | - | - | - | - | - | - | - |
| **feb_19** | **4.3E-02** | **4.1E-06** | 4.3E-01 | **1.9E-02** | **1.5E-04** | **7.9E-04** | **1.5E-03** | - | - | - | - | - | - | - | - | - | - | - | - | - | - | - | - | - | - | - |
| **feb_20** | 9.9E-02 | **9.5E-03** | **8.7E-05** | 3.7E-01 | **1.9E-08** | **1.9E-08** | 6.8E-01 | **2.1E-04** | - | - | - | - | - | - | - | - | - | - | - | - | - | - | - | - | - | - |
| **jan_19** | **1.3E-04** | **2.4E-07** | 3.8E-01 | **1.9E-03** | **8.1E-03** | 3.3E-01 | **8.8E-07** | **2.4E-02** | **2.2E-07** | - | - | - | - | - | - | - | - | - | - | - | - | - | - | - | - | - |
| **jan_20** | 6.1E-02 | 5.5E-02 | **3.3E-05** | 7.6E-01 | **2.3E-08** | **2.3E-08** | 5.8E-01 | **2.6E-04** | 8.8E-01 | **2.2E-07** | - | - | - | - | - | - | - | - | - | - | - | - | - | - | - | - |
| **jul_18** | **5.0E-05** | **1.9E-08** | 1.7E-01 | **1.6E-04** | **1.2E-02** | 9.0E-01 | **2.8E-07** | **1.2E-02** | **2.8E-07** | 3.5E-01 | **3.6E-08** | - | - | - | - | - | - | - | - | - | - | - | - | - | - | - |
| **jul_19** | 6.1E-01 | **4.2E-04** | 1.2E-01 | 7.2E-02 | **5.0E-05** | **2.0E-03** | 6.5E-02 | 4.3E-01 | **4.3E-02** | **1.7E-02** | **3.8E-02** | **6.2E-03** | - | - | - | - | - | - | - | - | - | - | - | - | - | - |
| **jul_20** | **4.3E-02** | **5.4E-04** | 7.5E-01 | **1.8E-02** | **3.8E-02** | 6.2E-01 | **6.0E-03** | 3.2E-01 | **3.2E-03** | 8.8E-01 | **2.3E-03** | 7.1E-01 | 1.8E-01 | - | - | - | - | - | - | - | - | - | - | - | - | - |
| **jun_18** | **1.4E-03** | **1.7E-07** | 7.8E-01 | **5.0E-03** | **1.8E-03** | 1.3E-01 | **5.0E-05** | 1.6E-01 | **5.9E-06** | 5.6E-01 | **2.1E-06** | 2.1E-01 | 6.3E-02 | 7.5E-01 | - | - | - | - | - | - | - | - | - | - | - | - |
| **jun_19** | 9.3E-01 | **4.6E-04** | **8.3E-03** | 1.1E-01 | **2.2E-07** | **3.6E-07** | 2.2E-01 | **2.0E-02** | 7.0E-02 | **1.5E-05** | 8.0E-02 | **1.6E-05** | 4.7E-01 | **2.5E-02** | **5.1E-04** | - | - | - | - | - | - | - | - | - | - | - |
| **jun_20** | 7.1E-01 | **4.3E-02** | 9.5E-02 | 2.6E-01 | **4.5E-04** | **4.0E-02** | 6.6E-01 | 2.7E-01 | 8.0E-01 | 1.0E-01 | 4.0E-01 | 5.0E-02 | 6.5E-01 | 2.7E-01 | 1.3E-01 | 7.6E-01 | - | - | - | - | - | - | - | - | - | - |
| **mar_19** | **2.9E-02** | **3.1E-06** | 6.3E-01 | **6.0E-03** | **1.9E-04** | **1.8E-03** | **1.1E-03** | 9.2E-01 | **1.0E-04** | **2.4E-02** | **1.1E-04** | **2.2E-02** | 3.2E-01 | 3.6E-01 | 1.9E-01 | **2.1E-02** | 1.4E-01 | - | - | - | - | - | - | - | - | - |
| **mar_20** | **4.9E-04** | 4.4E-01 | **2.2E-07** | 7.6E-01 | **9.4E-09** | **9.4E-09** | **3.5E-02** | **4.7E-07** | **2.5E-02** | **2.4E-08** | 1.3E-01 | **2.6E-08** | **4.3E-04** | **5.6E-04** | **1.9E-08** | **4.4E-04** | 1.3E-01 | **5.1E-07** | - | - | - | - | - | - | - | - |
| **may_19** | 4.6E-01 | **4.2E-04** | 2.0E-01 | 6.1E-02 | **2.0E-04** | **6.2E-03** | 6.3E-02 | 6.0E-01 | **3.8E-02** | **4.0E-02** | **2.5E-02** | **2.0E-02** | 8.0E-01 | 2.8E-01 | 1.5E-01 | 3.3E-01 | 5.2E-01 | 4.6E-01 | **5.1E-04** | - | - | - | - | - | - | - |
| **may_20** | 4.6E-02 | **5.4E-02** | **3.3E-05** | 6.8E-01 | **1.9E-07** | **2.2E-07** | 4.6E-01 | **8.0E-04** | 5.3E-01 | **3.1E-06** | **7.4E-01** | **5.8E-06** | **2.7E-02** | **9.6E-03** | **3.9E-05** | 5.7E-02 | 5.0E-01 | **3.8E-04** | 2.6E-01 | **3.0E-02** | - | - | - | - | - | - |
| **nov_18** | **1.6E-02** | **3.7E-06** | 6.7E-01 | **1.1E-02** | **1.1E-04** | **3.0E-03** | **3.6E-04** | 7.6E-01 | **3.4E-05** | **4.4E-02** | **7.2E-05** | **2.2E-02** | 2.8E-01 | 3.2E-01 | 2.6E-01 | **8.0E-03** | 2.5E-01 | 7.6E-01 | **5.6E-07** | 4.6E-01 | **2.3E-04** | - | - | - | - | - |
| **nov_19** | 3.6E-01 | **6.1E-05** | 8.5E-02 | **3.8E-02** | **1.8E-06** | **1.8E-05** | **2.3E-02** | 1.6E-01 | **4.7E-03** | **8.0E-04** | **5.9E-03** | **2.2E-04** | 9.5E-01 | 7.1E-02 | **1.1E-02** | 2.9E-01 | 4.8E-01 | 2.0E-01 | **3.9E-05** | 8.0E-01 | **1.2E-02** | 1.2E-01 | - | - | - | - |
| **oct_18** | 5.0E-02 | 6.8E-02 | **3.4E-05** | 7.8E-01 | **1.9E-08** | **2.2E-08** | 5.1E-01 | **2.4E-04** | 5.7E-01 | **2.2E-07** | 7.8E-01 | **4.7E-07** | **2.0E-02** | **3.3E-03** | **9.9E-06** | **3.8E-02** | 4.6E-01 | **1.5E-04** | 2.3E-01 | **1.3E-02** | 8.5E-01 | **7.2E-05** | 5.0E-03 | - | - | - |
| **oct_19** | **3.5E-04** | **3.0E-07** | 3.5E-01 | **5.4E-04** | **7.1E-03** | 6.8E-01 | **3.5E-06** | **2.4E-02** | **7.7E-07** | 5.3E-01 | **5.4E-07** | 6.9E-01 | **1.3E-02** | 9.2E-01 | 3.6E-01 | **4.3E-05** | 7.2E-02 | **3.8E-02** | **2.4E-08** | **3.3E-02** | **2.7E-06** | **5.0E-02** | **1.4E-03** | **5.4E-07** | - | - |
| **sep_19** | 1.4E-01 | **1.6E-04** | 4.9E-01 | **3.2E-02** | **2.6E-04** | **1.1E-02** | **9.5E-03** | 8.0E-01 | **3.3E-03** | 5.2E-02 | **3.0E-03** | **4.0E-02** | 5.1E-01 | 3.2E-01 | 2.6E-01 | 9.6E-02 | 4.4E-01 | 8.0E-01 | **1.5E-04** | 7.4E-01 | **7.9E-03** | 7.6E-01 | 4.4E-01 | **3.1E-03** | 7.1E-02 | - |
| **sep_18** | 2.5E-01 | **1.9E-02** | **1.5E-03** | 4.7E-01 | **3.8E-07** | **9.6E-06** | 9.4E-01 | **1.3E-02** | 8.8E-01 | **1.5E-04** | 5.8E-01 | **5.8E-06** | 1.7E-01 | **1.3E-02** | **3.2E-04** | 3.1E-01 | 7.5E-01 | **3.9E-03** | **4.5E-02** | 1.2E-01 | 4.6E-01 | **2.9E-03** | **4.4E-02** | 4.8E-01 | **7.7E-05** | **3.0E-02** |

**Table S1** Pairwise comparisons between each single month available over the three years of observation, obtained by the Wilcoxon rank sum test. (In **bold** p-value < 0.05 while in grey p-value >0.05).

|  | **autumn_18** | **autumn_19** | **spring_19** | **spring_20** | **summer_18** | **summer_19** | **summer_20** | **winter_19** |
| --- | --- | --- | --- | --- | --- | --- | --- | --- |
| **autumn_19** | **9.10E-05** | - | - | - | - | - | - | - |
| **spring_19** | **3.07E-02** | 6.60E-02 | - | - | - | - | - | - |
| **spring_20** | **9.40E-06** | **7.90E-14** | **1.10E-10** | - | - | - | - | - |
| **summer_18** | **6.10E-09** | **3.91E-02** | **1.10E-04** | **2.00E-16** |  | - | - | - |
| **summer_19** | 8.75E-01 | **1.60E-04** | **2.33E-02** | **1.20E-04** | **5.50E-08** | - | - | - |
| **summer_20** | **5.70E-06** | **2.03E-02** | **2.23E-03** | **8.90E-11** | 3.23E-01 | **8.80E-06** | - | - |
| **winter_19** | **5.50E-10** | **2.03E-02** | **8.20E-06** | **2.00E-16** | 9.33E-01 | **1.20E-08** | 2.87E-01 | - |
| **winter_20** | **2.86E-02** | **2.30E-10** | **5.40E-06** | **2.29E-03** | **7.60E-16** | 8.25E-02 | **5.90E-09** | **2.00E-16** |

**Table S2** Pairwise comparisons between each single season available over the three years of observation, obtained by the Wilcoxon rank sum test. (In **bold** p-value < 0.05 while in grey p-value >0.05).


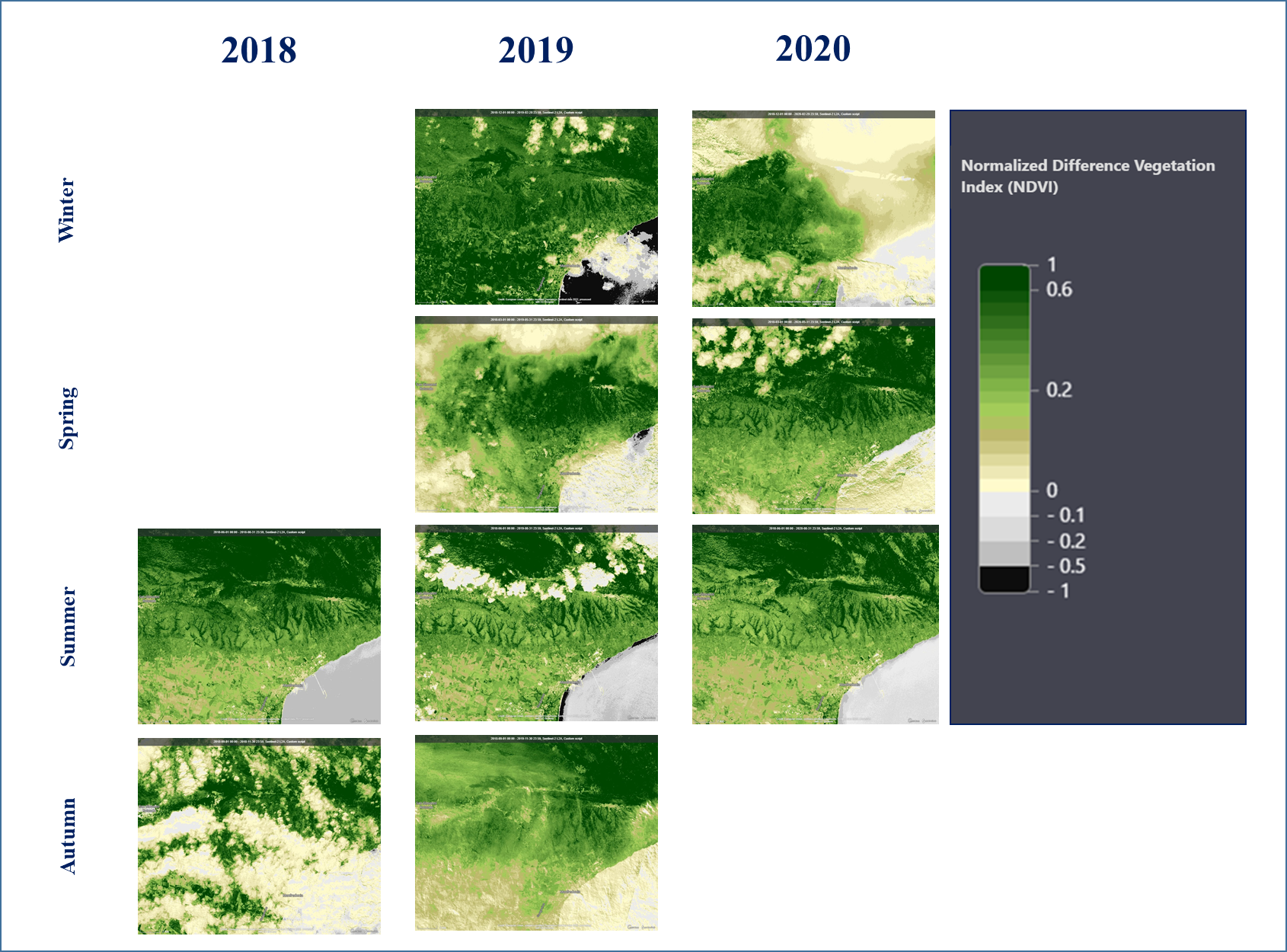


**Fig. S1.** Normalized Difference Vegetation Index (NDVI) seasonal maps providing a measure of vegetation density and condition in the area surrounding MSA sampling station. This index is based on satellite data from the Copernicus Sentinel 2 mission (https://apps.sentinel-hub.com).


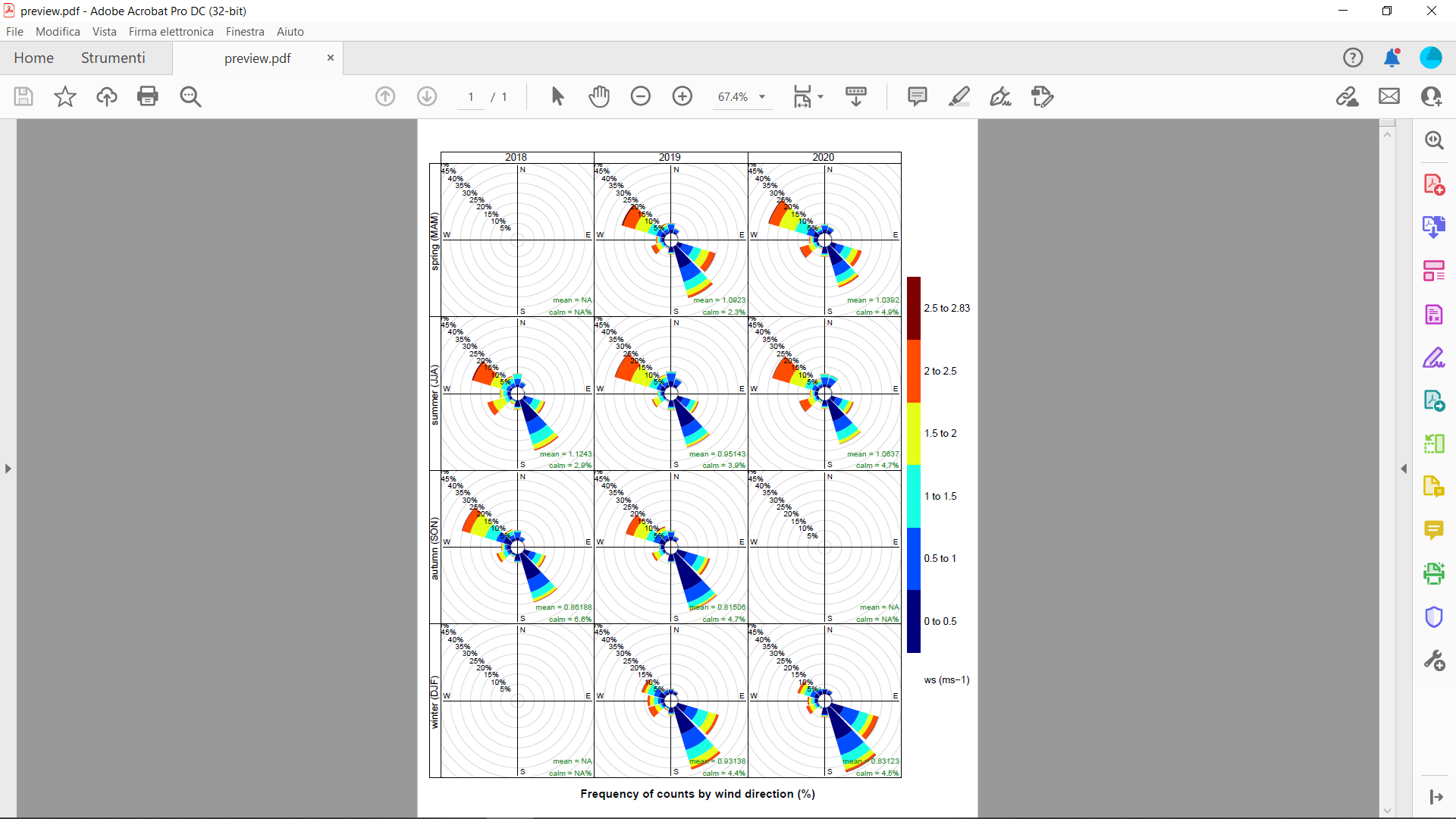

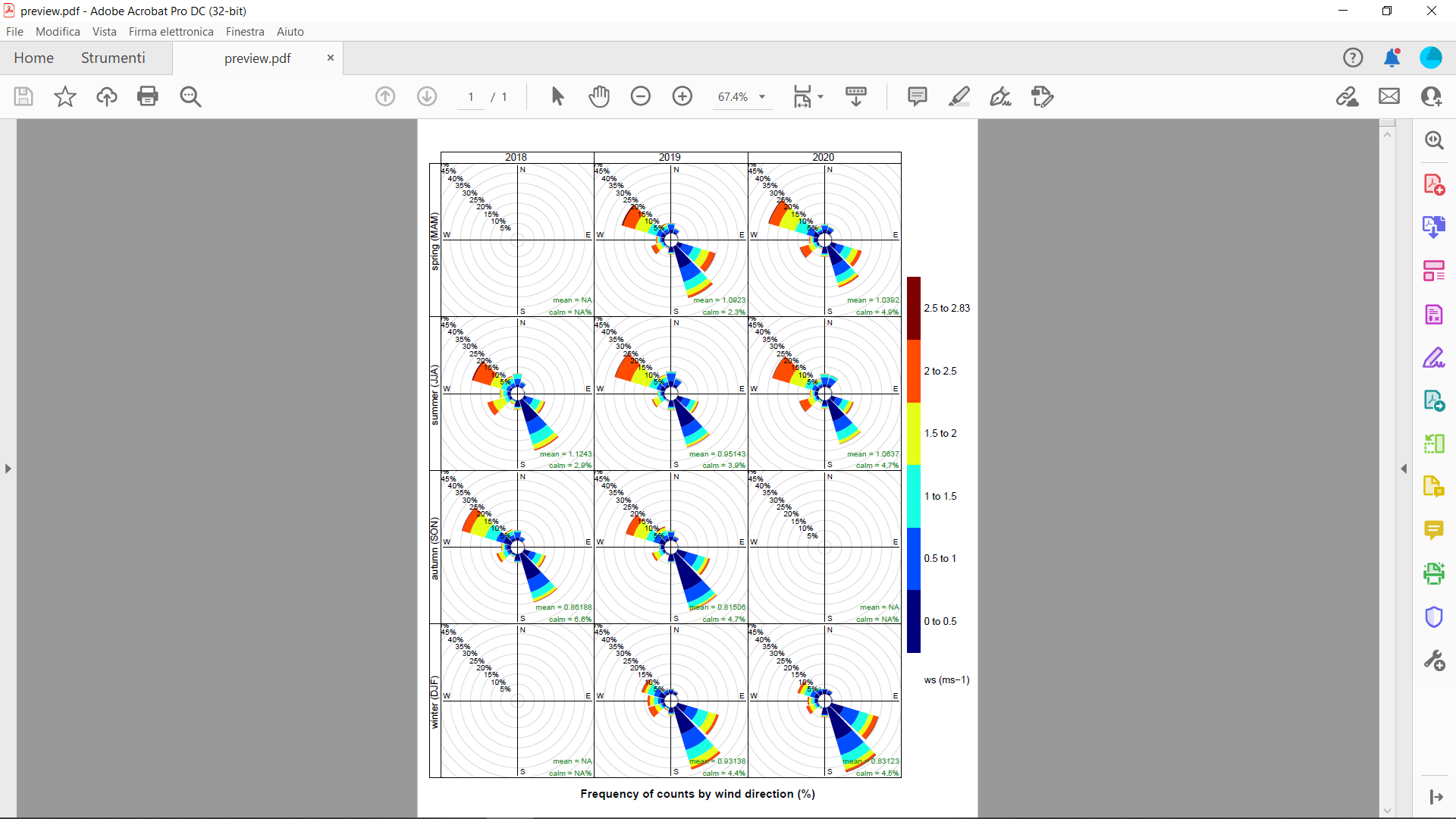

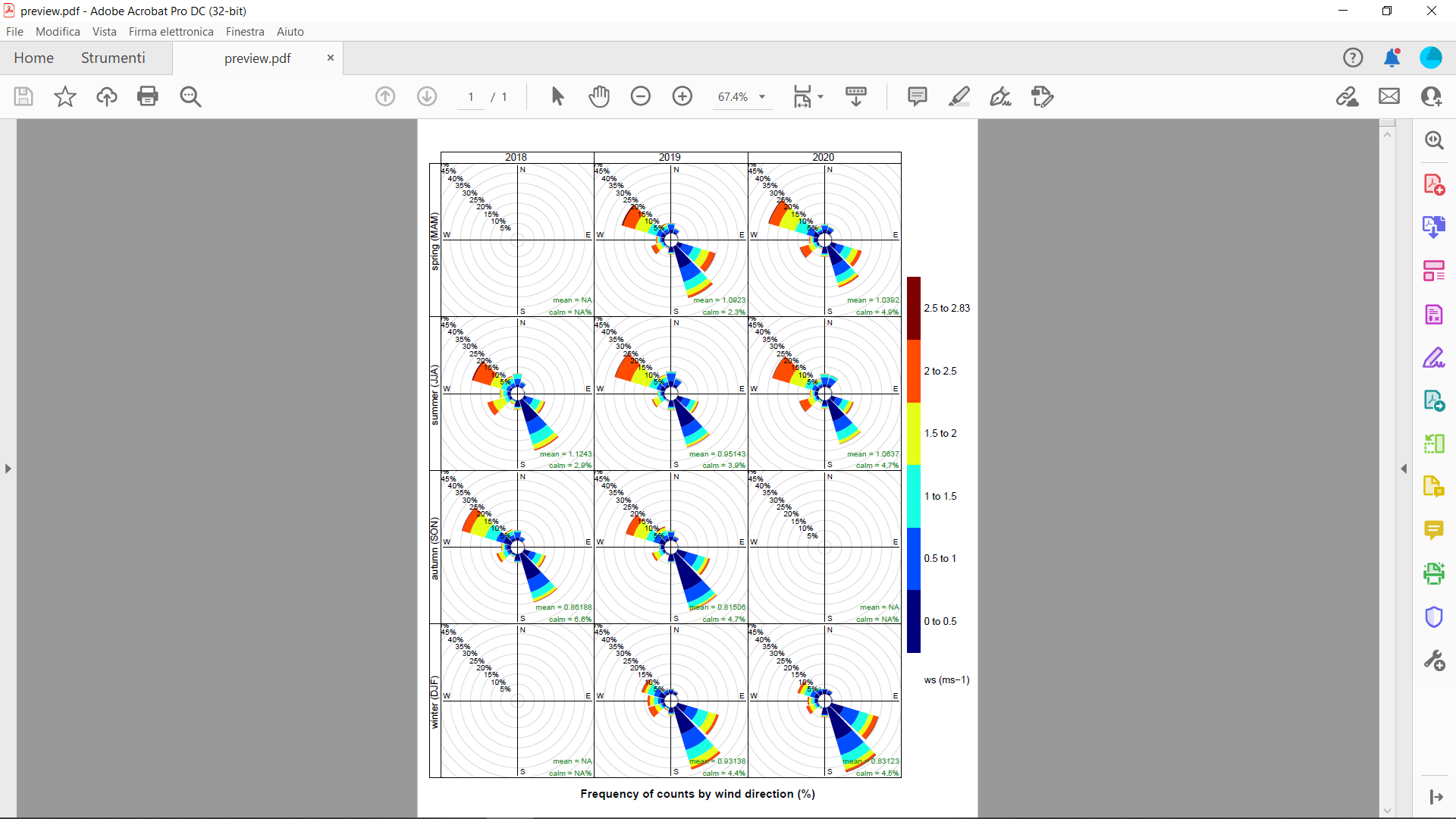

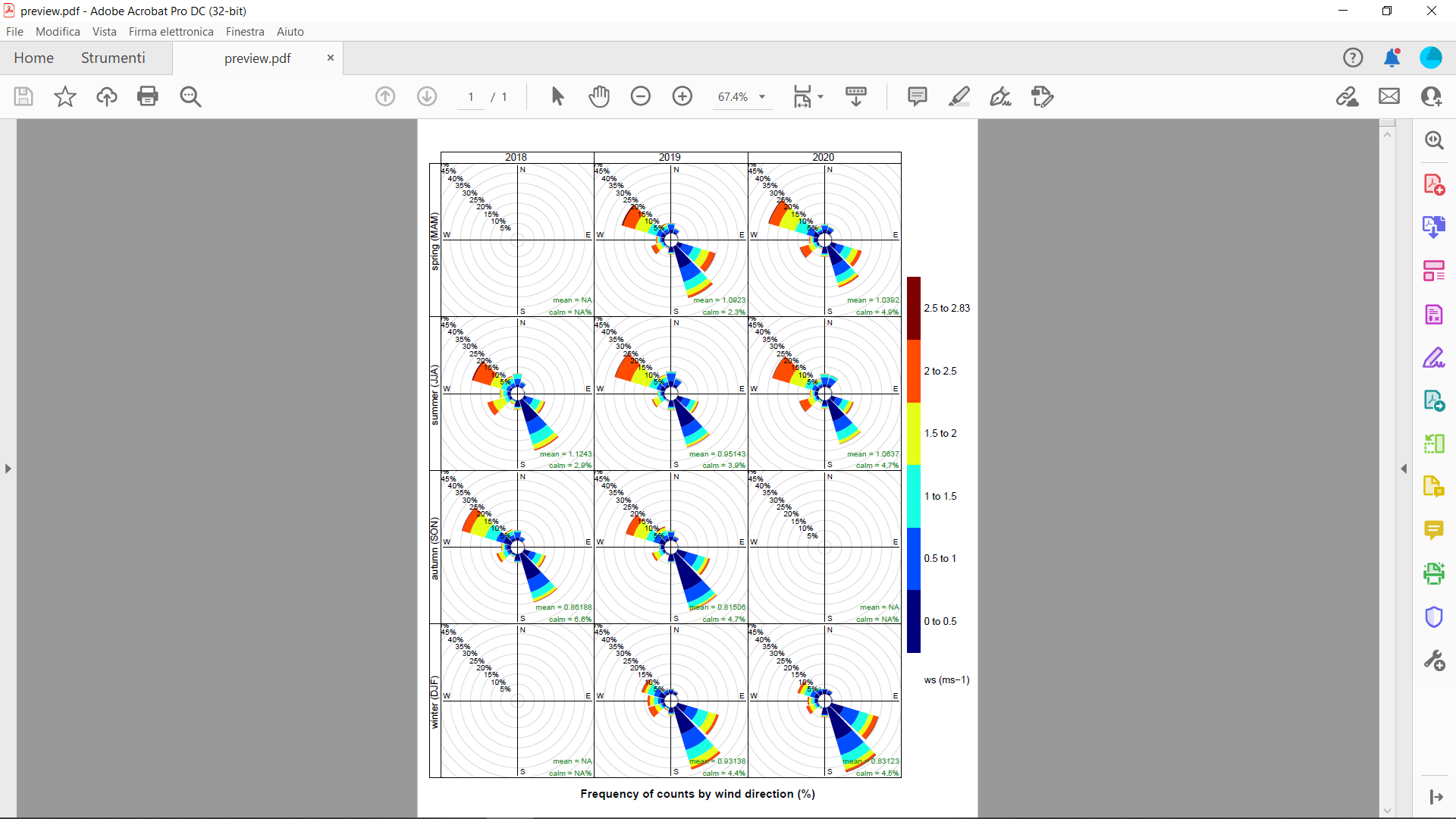

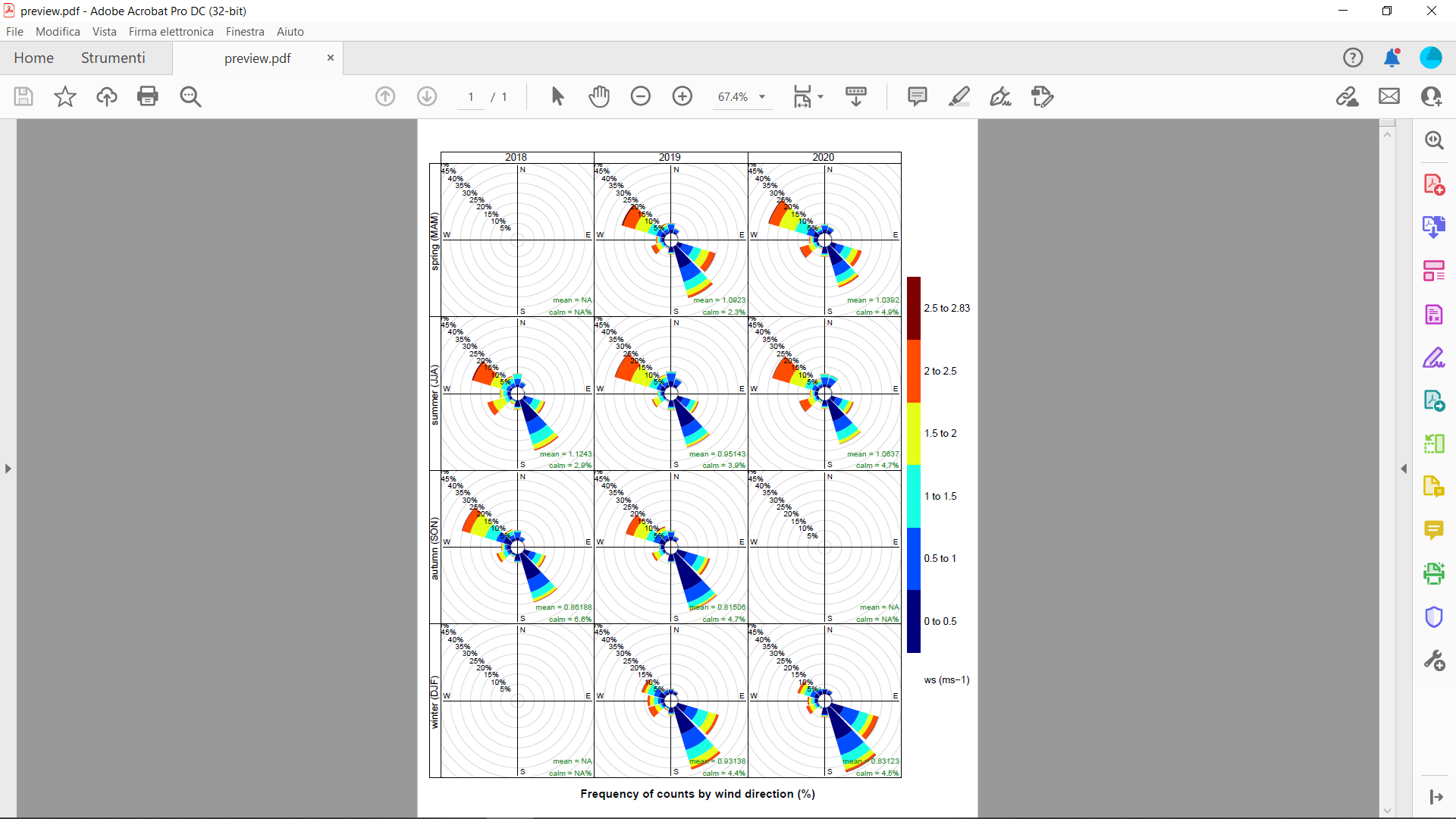


**Fig. S2.** Wind roses, with the wind speed/direction frequencies, by each season over the available years.

**Fig. S3** Map of kriged residuals including outliers. Overlaid are coal fired power plants in circles with the size of the circle proportional to the power production and Hg mines with the size of the symbols representing their past production (*Source*: (Ballabio et al. 2021) - <https://doi.org/10.1016/j.scitotenv.2020.144755>).


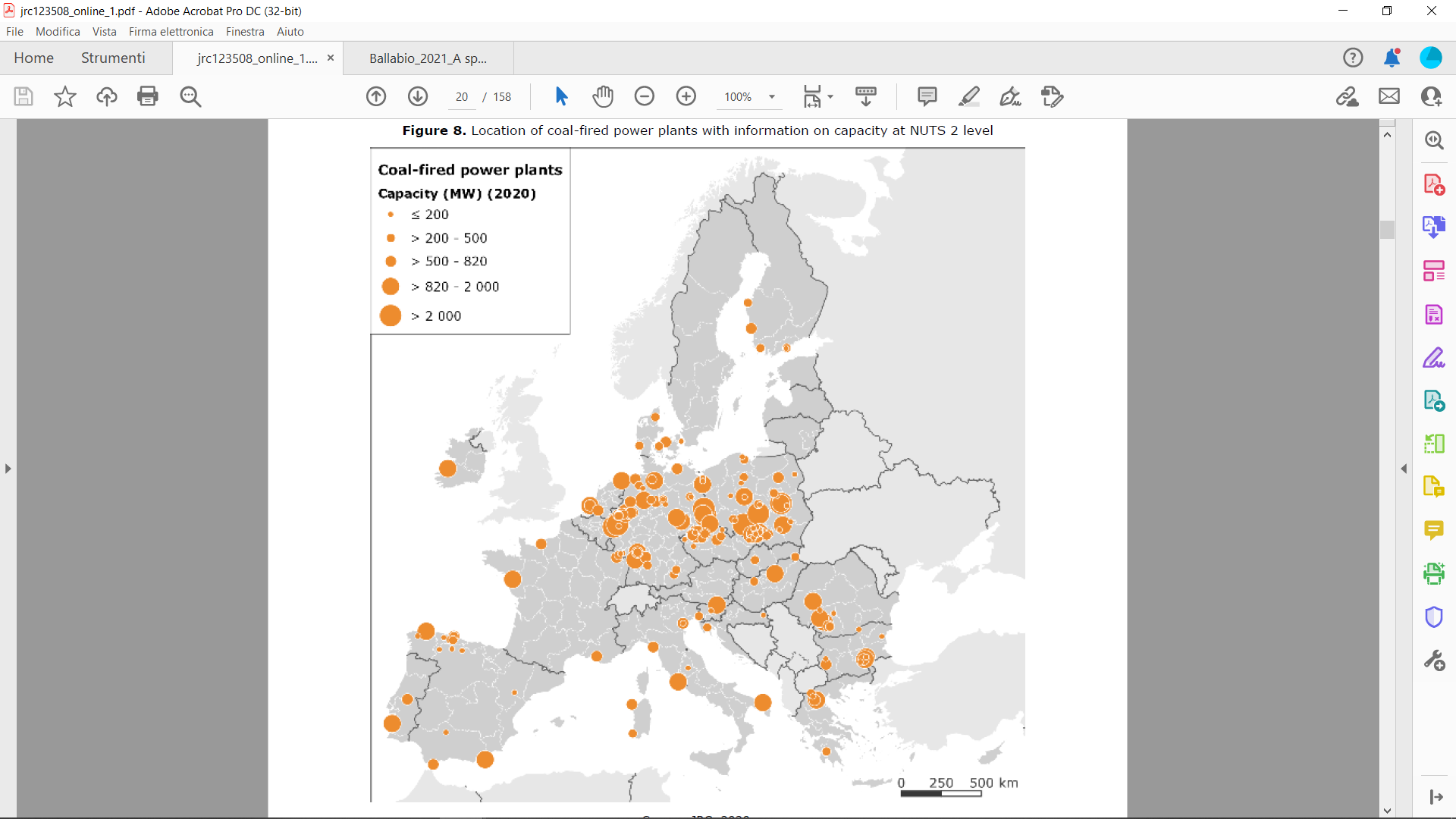


**Fig. S4** Location of coal-fired power plants with information on capacity

(Source: (Kapetaki et al. 2021) - <https://ec.europa.eu/jrc/sites/default/files/factsheet_on_eu_trends_coal_peat_oil.pdf> ).


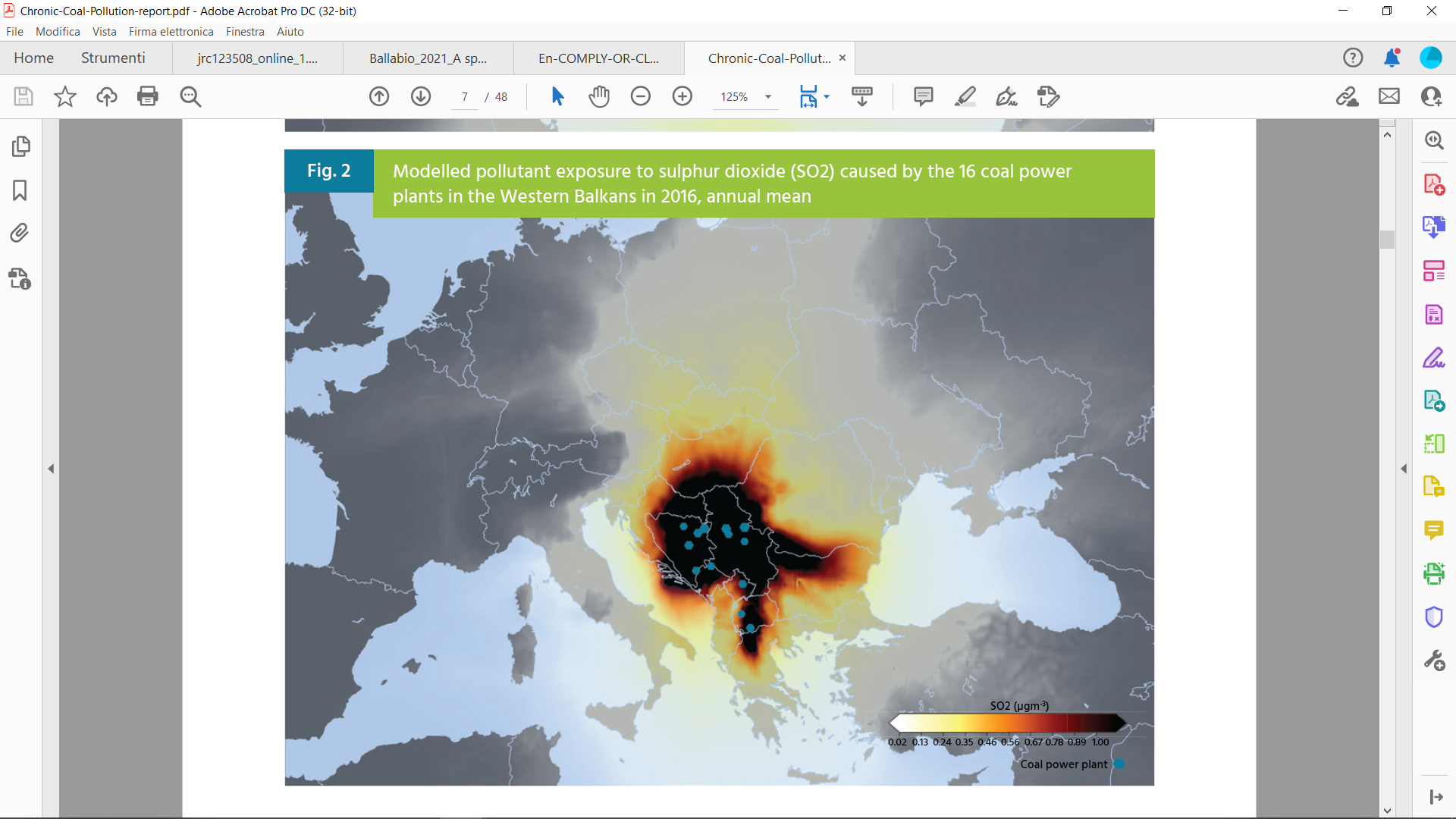


**Fig. S5** Modelled pollutant exposure to solphur dioxide (SO_2_) caused by the 16 coal power plants in the Western Balkans in 2016, annual mean. (Source: (Jensen, GK et al., 2019) - <https://www.env-health.org/wp-content/uploads/2019/02/Chronic-Coal-Pollution-report.pdf> ).


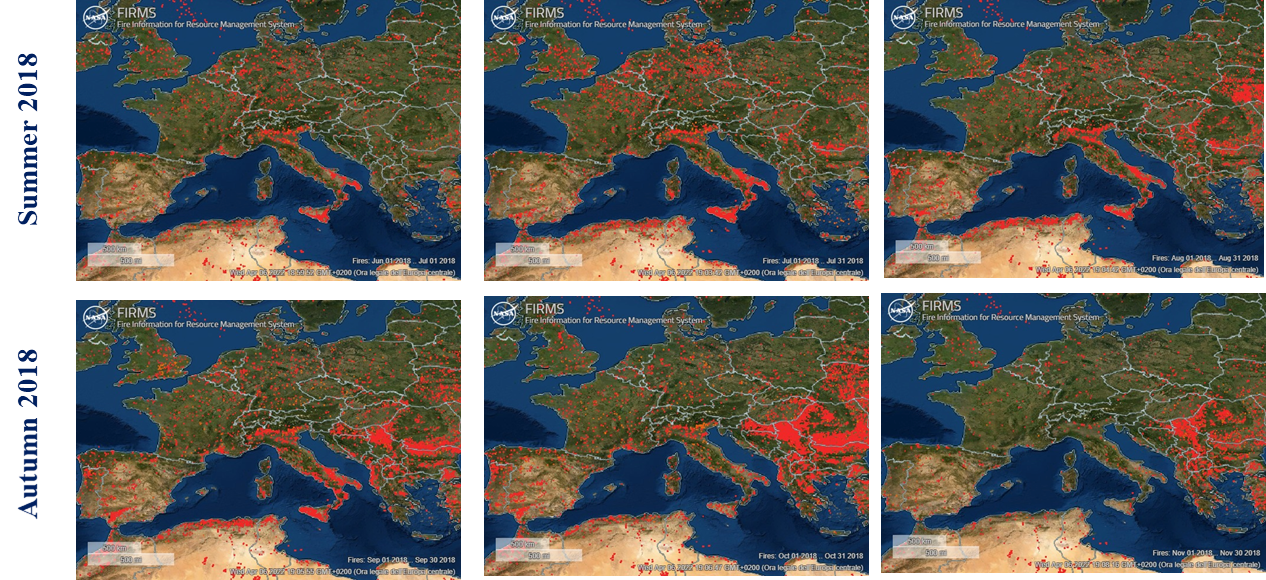


**Fig. S6.** FIRMS maps showing monthly cumulative active hot-spot fires over Europe during Summer and Autumn 2018.


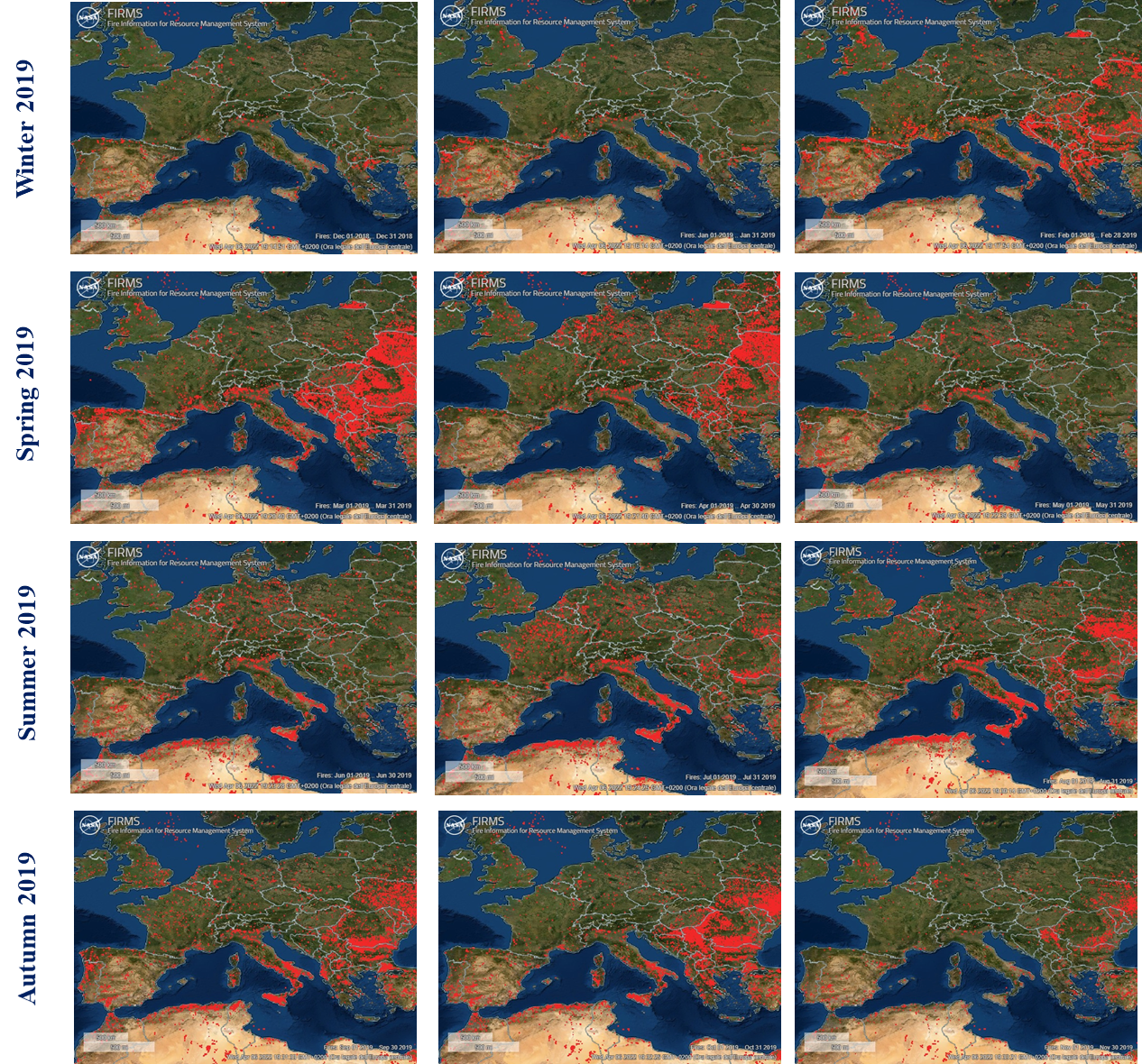


**Fig. S7** FIRMS maps showing monthly cumulative active hot-spot fires over Europe during Winter, Spring, Summer, and Autumn 2019.


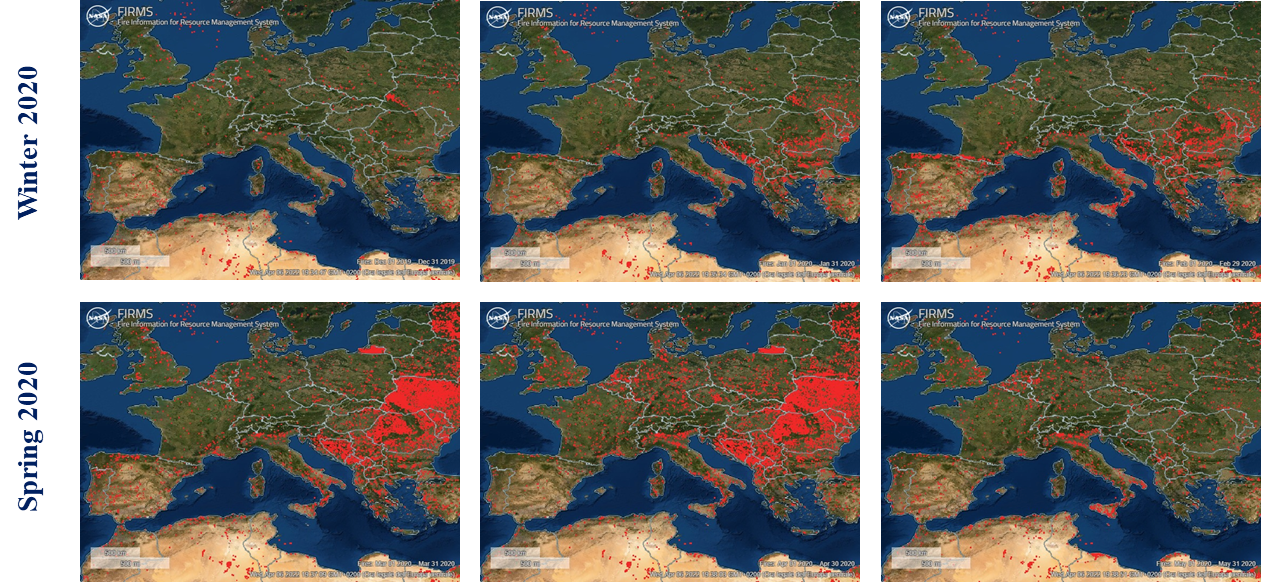


**Fig. S8** FIRMS maps showing monthly cumulative active hot-spot fires over Europe during Winter and Spring 2020.


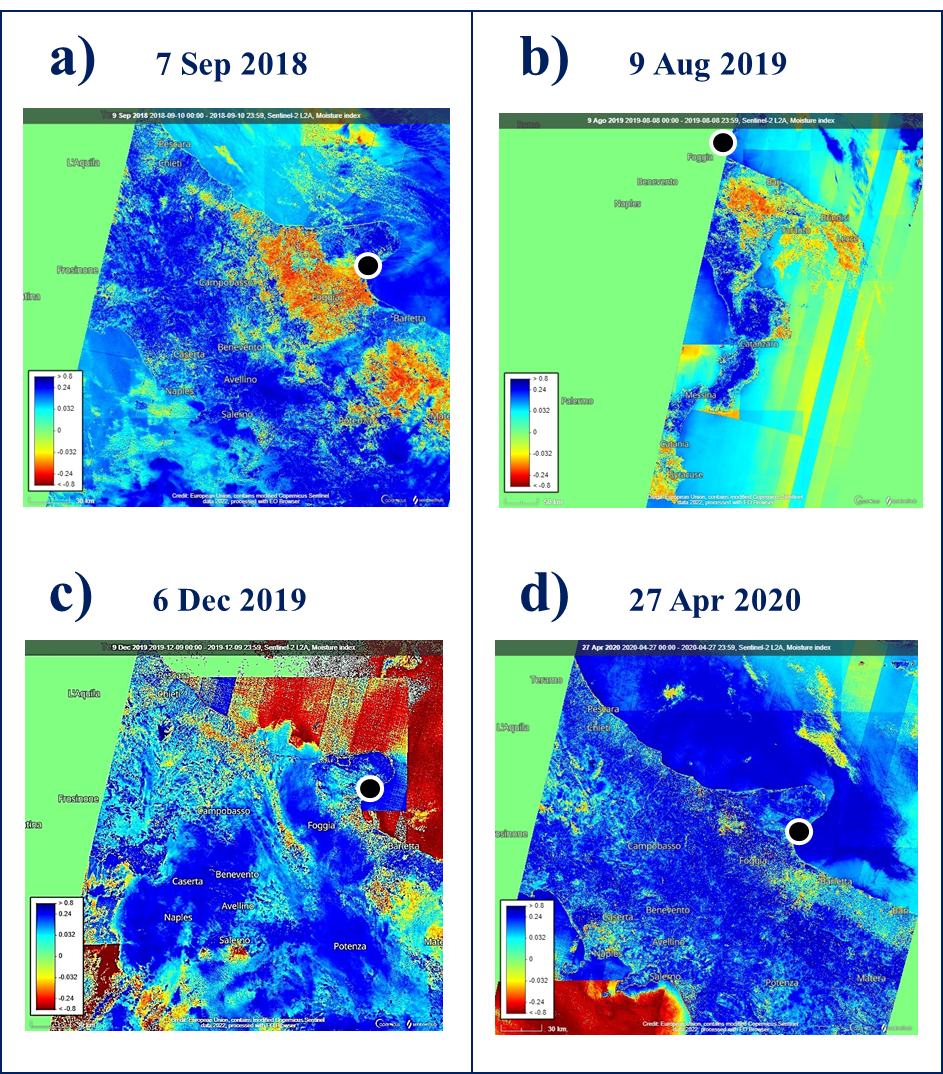


**Fig. S9** Normalized Difference Moisture Index (NDMI), supported by Sentinel-2, providing indication about the vegetation water stress for the following selected days: a) 7 September 2018, b) 9 August 2019, c) 6 December 2019, and d) 27 April 2020.

**References**

Ballabio C, Jiskra M, Osterwalder S, et al (2021) A spatial assessment of mercury content in the European Union topsoil. Sci Total Environ 769:144755. <https://doi.org/10.1016/j.scitotenv.2020.144755>

Kapetaki Z, Alves Dias P, Conte A, et al (2021) Recent trends in EU coal, peat and oil shale regions. Publications Office of the European Union. <https://ec.europa.eu/jrc/sites/default/files/factsheet_on_eu_trends_coal_peat_oil.pdf>

Chronic coal pollution - EU action on the Western Balkans will improve health and economies across Europe. HEAL, CAN Europe, Sandbag, CEE Bankwatch Network and Europe Beyond Coal. 2019. <https://www.env-health.org/wp-content/uploads/2019/02/Chronic-Coal-Pollution-report.pdf>
